# Genome-wide identification and analysis of the evolution and expression pattern of the *HVA22* gene family in three wild species of tomatoes

**DOI:** 10.7717/peerj.14844

**Published:** 2023-02-13

**Authors:** LaiPeng Zhao, Baike Wang, Tao Yang, Huizhuan Yan, Qinghui Yu, Juan Wang

**Affiliations:** 1 Institute of Horticultural Crops, Xinjiang Academy of Agricultural Science (Key Laboratory of Horticulture Crop Genomics Research and Genetic Improvement in Xinjiang), Urumqi, Xinjiang, China; 2 College of Horticulture, Xinjiang Agricultural University, Urumqi, Xinjiang, China

**Keywords:** *HVA22*, Bioinformatics, Phylogenetic analysis, Abiotic stress, Wild tomato

## Abstract

Wild tomato germplasm is a valuable resource for improving biotic and abiotic stresses in tomato breeding. The *HVA22* is widely present in eukaryotes and involved in growth and development as well as stress response, such as cold, salt, drought, and biotic stress. In the present study, we identified 45 *HVA22* genes in three wild species of tomatoes. The phylogenetic relationships, gene localization to chromosomes, gene structure, gene collinearity, protein interactions, and *cis*-acting element prediction of all 45 *HVA22* genes (14 in *Solanum pennellii,* 15 in *S. pimpinellifolium,* and 16 in *S. lycopersicoides*) were analyzed. The phylogenetic analysis showed that the all HVA22 proteins from the family Solanaceae were divided into three branches. The identified 45 *HVA22* genes were grouped into four subfamilies, which displayed similar number of exons and expanded in a fragmentary replication manner. The distribution of *HVA22* genes on the chromosomes of the three wild tomato species was also highly similar. RNA-seq and qRT-PCR revealed that *HVA22* genes were expressed in different tissues and induced by drought, salt, and phytohormone treatments. These results might be useful for explaining the evolution, expression patterns, and functional divergence of *HVA22* genes in *Lycopersicon*.

## Introduction

The *HVA22* gene was first isolated from the dextrin layer of barley (*Hordeum vulgare*) in 1993 (Shen, Uknes & Ho, 1993). Homologs to the *HVA22* gene have been identified in eukaryotes, such as yeast, cereals, *Arabidopsis thaliana*, nematodes, mice, and humans. Approximately 367 *HVA22* homologs have been found in eukaryotes, which present a conserved TB2/DP1 (deleted-in-polyposis) domain (PF03134) (Guo & David Ho, 2008; Sharon & Suvarna, 2017; Gomes Ferreira et al., 2019). Notably, no homologs have been described in prokaryotes to date, suggesting that *HVA22* is likely involved in eukaryote-specific functions.

In plants, the expression patterns of *HVA22* during development are well characterized. The accumulated transcripts of *HVA22* homologs in leaves are highly induced by abscisic acid (ABA), drought, cold, and salt stresses in both barley and *A. thaliana* (Shen, Uknes & Ho, 1993; Shen et al., 2001; Chen et al., 2002). The heterologous expression of the barley *HVA22* protein improves the salt tolerance and the survival rate of *E. coli* under cold stress by delaying its proliferation (Lu, 2013). Additionally, a recent study on barley discovered that the amount of the transcription product of the *HVA22* gene was significantly higher in barley *hvabi5d* mutant strains than in wild-type plants under drought conditions (Collin et al., 2020). Subsequently, the *HVA22* gene was also shown to be induced by drought and salt stresses in different plants. For example, the accumulation of transcript of *HVA22* homologs in common wheat roots under drought stress was reported (Grzesiak et al., 2019). The expression of the *HVA22* gene was significantly higher in drought-tolerant bermudagrass subjected to external simulated drought (Liu et al., 2014). The HVA22-like protein encoded by BG598159 was found to play an important role in salt and drought stress in potato by interacting with the StPDI1 protein and participating in the sucrose transport pathway (Eggert et al., 2016). The expression of the tomato *HVA22* gene was also significantly induced by salt and drought stresses (Wai et al., 2022). Also, the heterologous expression of the *Citrus clementina CcHVA22d* gene in tobacco enhanced dehydration tolerance and significantly reduced the H_2_O_2_ content in a short-term dehydration environment (Gomes Ferreira et al., 2019).

Studies have shown that the *HVA22* gene also plays an important role in endoplasmic reticulum–related pathways. The Yop1p gene, a homolog of *HVA22* in yeast, appears to be involved in the translocation of substances from the endoplasmic reticulum to the Golgi apparatus during cellular activity (De Antoni et al., 2002). Previous studies on the yeast Yop1p protein revealed that the Yop1p/DP1 protein interacted with the Rtn4/NogoA protein, thereby co-regulating the interactions between other proteins *in vivo* as well as endoplasmic reticulum function (Hu et al., 2008). The *HVA22* gene in the dextrin layer of barley seeds also has a similar function as yeast Yop1p. The accumulation of the *HVA22* gene in the dextrin layer after induction by abscisic acid (ABA) inhibits vesicle transport in cells, thereby delaying the incorporation of storage protein vesicles, which is a process thought to play a role in regulating seed germination and seedling growth (Guo & David Ho, 2008). More recent studies in rice have shown that the rice *HVA22* family gene *OsHLP1* promotes disease resistance mechanisms in plants by maintaining endoplasmic reticulum homeostasis (Meng et al., 2022). To date, studies on the involvement of *HVA22* homologs in the cellular vesicle transport pathway are scarce.

The supply of food and vegetable production have become major issues with the continuous rise in the world population. This is compounded by the potential impact of an increasingly changing climate on crop productivity. Extreme temperatures, drought, and soil salinization are the main adverse environments often encountered by plants (Gong et al., 2020). Tomatoes are a favorite vegetable worldwide. However, adverse environments such as salt, drought, and cold severely affect tomato growth and development (Chaudhary et al., 2019a; Chaudhary et al., 2019b). Wild tomatoes belonging to the genus *Lycopersicon* have higher tolerance to salt, drought, and cold than cultivated tomatoes (Szymański et al., 2020). Thus, wild tomatoes were an important genetic resource for our study on tomato response to adversity. It has been demonstrated that *HVA22* genes are significantly upregulated in rice (Zhao et al., 2021), *A. thaliana* (Chen et al., 2002), barley (Shen et al., 2001), and tomatoes (Wai et al., 2022) in response to salt and drought stresses. However, systematic studies on *HVA22* family genes in wild tomatoes have not been reported. In this study, we used the bioinformatics methods to comprehensively identify *HVA22* family genes in three species of wild tomatoes (*S. pimpinellifolium*, *S. pennellii*, and *S. lycopersicoides*). This study might provide a theoretical reference for elucidating *HVA22* family gene members and mining tomato genes for resistance to abiotic stresses.

## Materials & Methods

### Identification of *HVA22* family genes in the family Solanaceae

The protein sequence of the *Arabidopsis thaliana HVA22* family gene was downloaded from the Ensembl database (http://plants.ensembl.org/index.html) (Yates et al., 2022). Protein sequence files for three species of wild tomatoes (*S. pimpinellifolium*, *S. pennellii*, and *S. lycopersicoides*), tobacco (*Nicotiana benthamiana*), pepper (*Capsicum annuum*), eggplant (*Solanum melongena*), and potato (*Solanum tuberosum*), as well as genome files from the Solanaceae genome database (https://Solgenomics.net/), were downloaded (Fernandez-Pozo et al., 2015). The hidden Markov model of the structural domain of the HVA22-like protein TB2/DP was obtained from the Pfam (PF03134) (http://pfam.xfam.org/) and PANTHER (PTHR12300) (http://www.pantherdb.org/) database (Mistry et al., 2021; Thomas et al., 2022). The screened *HVA22* protein sequences were validated using the online protein structural domain prediction tool HMM search (http://hmmer.org/) (Finn, Clements & Eddy, 2011), and genes that did not contain the TB2/DP structural domain were removed. The physicochemical properties of the screened tomato *HVA22* family of proteins were predicted on the ExPASy website (https://www.expasy.org/protparam/) (Artimo et al., 2012). The subcellular localization prediction of *HVA22* family genes in three species of wild tomatoes was performed on the WoLF PSORT online tool (https://wolfpsort.hgc.jp/) (Horton et al., 2007).

### Construction of conserved motifs, *cis*-*acting* elements, and phylogenetic tree of *HVA22* gene in three species of wild tomatoes

The *HVA22* family gene motifs (Grundy et al., 1997) in the three species of wild tomatoes were searched using the MEME online tool (https://meme-suite.org/meme/tools/meme). The number of search base sequences was set to 20, and the minimum and maximum widths were set to 6 and 50, respectively. The results from the MEME search were used to map the conserved modal motifs and gene structures using TBtools. Multiple sequence comparisons were performed using MEGA 11 software, and a phylogenetic tree was constructed using the maximum likelihood method (ML) (Tamura, Stecher & Kumar, 2021). The constructed phylogenetic trees were embellished using the online tools ITOOL (https://itol.embl.de/) (Letunic & Bork, 2021). The 2,000-bp promoter sequence upstream of the *HVA22* family gene in the three species of wild tomatoes was extracted, and the *cis-acting* element of the *HVA22* family gene was predicted using the PlantCARE database (http://bioinformatics.psb.ugent.be/webtools/plantcare/html/) (Rombauts et al., 1999) and visualized using TBtools (Chen et al., 2020).

### Interaction network and expression analysis of *HVA22* family homologous genes

MCScanX was used to analyze the *HVA22* gene in four species of *Lycopersicon* (*S. lycopersicum*, *S. lycopersicoides*, *S. pennellii*, and *S. pimpinellifolium*) and Solanaceae [tobacco (*N. benthamiana*), pepper (*C. annuum*), potato (*S. tuberosum*) and eggplant (*S. melongena*)] interspecies as well as the intraspecific collinearity in four species of *Lycopersicon*. The substitution rate of paralogous homologous genes was calculated using Ka/Ks_Calculator 2.0 (Wang et al., 2010). The direct homologous genes between species and the paralogous homologous gene collinearity within species were visualized using TBtools. The STRING online website was used to predict protein–protein interaction relationships (Szklarczyk et al., 2019), interactions, after which the relationship data given by the predictions were visualized using Cytoscape 3.9.1. The expression matrices for different tissues and developmental stages of *S. pimpinellifolium* were downloaded from the Tomato Function Genomics database (http://ted.bti.cornell.edu/), which included expression data for root, stem, leaves, young flower buds, anthesis flowers, 10 days post anthesis, 20 days post anthesis, 30 days post anthesis, and ruptured fruit (Fei et al., 2010). From these, *HVA22* family genes were selected and the expression profiles were heat-mapped using TBtools.

### Total RNA extraction and reverse transcription

The extraction of plant leaf RNA was accomplished using a Tiangen plant polyphenol polysaccharide total RNA extraction kit (Beijing, China). The cDNA synthesis of extracted total RNA was performed using a 5 × All-ln-one RTMasterMix (AccuRT Genomic DNA Removal Kit; G492, ABM, Vancouver, Canada) reverse transcription kit. qRT-PCR-specific primers were designed using the NCBI online primer tool (https://www.ncbi.nlm.nih.gov/tools/primer-blast/index.cgi?LINK_LOC=BlastHome), and the designed qRT-PCR primers were sent to Biotech Biologicals (Shanghai, China) for synthesis (Table S1). Quantitative PCR (qPCR) analysis was subsequently performed on a LightCycler machine using ChamQ Universal SYBR qPCR Master Mix (Q711, Vazyme, Nanjing, China) with *Slactin* as the internal reference gene. Three replicates of each treatment were performed. The relative expression was calculated by the 2^−ΔΔCt^ Method (Livak & Schmittgen, 2001).

### Plant material and growing conditions

The plant material used in this study was a wild tomato variety (*S. pimpinellifolium*, LA1589). For stress treatment, the seeds were sown in nutrient soil and vermiculite (*v*/*v* = 2:1) in a growth room at 24 ± 2 °C under 16-h light /8-h dark cycle. The seedlings were treated with Hoagland every week until use. Then, the plants were treated with different stresses, including 100 mM abscisic acid (ABA) or methyl jasmonate (MeJA), 200 mM NaCl, and 15% PEG6000. The leaves were collected and stored in liquid nitrogen quickly for RNA extraction at different time points (0, 2, 6, 12, and 24 h). Three independent biological replicates were included for each sample in the experiment.

## Results

### Identification of physicochemical properties and prediction of subcellular localization of *HVA22* family proteins from the three species of wild tomatoes

In the present study, we used the Simple HMM Search function of the TBtools tool to search the *HVA22* gene in *S. lycopersicoides*, *S. pennellii*, and *S. pimpinellifolium*. The retrieved genes were then subjected to structural domain validation using the HMM search (http://hmmer.org/) (Finn, Clements & Eddy, 2011) and InterPro (https://www.ebi.ac.uk/interpro/result/InterProScan/#table) online tool, and those without TB2/DP1 structural domains were discarded. The 45 *HVA22* family genes were finally determined in three species of wild tomatoes (15 in *S. pimpinellifolium*, 16 in *S. lycopersicoides*, and 14 in *S. pennellii*) and named as *HVA22a–HVA22p* according to their positions on chromosomes. Subsequently, we characterized the physicochemical properties and predicted the subcellular localization of the *HVA22* gene family member proteins from these three species of wild tomatoes. All *HVA22* family proteins in three species of wild tomatoes (15 in *S. pimpinellifolium* 14 in *S. pennellii*, and 16 in *S. lycopersicoides*) had amino acid lengths between 88 and 603 and protein molecular masses between 9989.65 to 68493.8 Da, with an isoelectric point of 5.53–10.09 and aliphatic index of 51.68–123.66. In terms of hydrophilicity, most of the proteins exhibited hydrophilic proteins (GRAVY <0) and a few exhibited hydrophobic proteins (GRAVY >0). The subcellular localization predictions showed that HVA22-like was localized to multiple organelles in three species of wild tomatoes; most *HVA22* family genes were localized to the endoplasmic reticulum, chloroplasts, cytoplasm, and nucleus; only the *SpiHVA22k* gene was localized to the vesicle (Table 1).

**Table 1:** Physicochemical properties of three species from wild tomato HVA22 protein.

**Species**	**Gene id**	**Gene name**	**Length**	**MW(Da)**	**pI**	**Aliphatic index**	**GRAVY**	**Subcellular localization**
*S.pimpinellifolium*	Spim05g006320.1.1	*SpiHVA22h*	171	20248.41	7.67	98.71	−0.101	E.R
	Spim03g025310.1.1	*SpiHVA22c*	182	20623.89	6.65	102.86	0.066	Chlo
	Spim06g022990.1.1	*SpiHVA22i*	133	15554.63	9.49	115.11	0.249	Extr
	Spim06g026920.1.1	*SpiHVA22j*	135	15665.57	8.8	104.74	0.21	Chlo
	Spim11g010400.1.1	*SpiHVA22o*	167	19963.73	9.32	113.23	0.34	Chlo
	Spim03g033180.1.1	*SpiHVA22d*	176	20507.66	6.41	96.93	0.005	Extr
	Spim04g011050.1.1	*SpiHVA22e*	105	12446.7	8.66	104.86	0.13	Cyto
	Spim10g007900.1.1	*SpiHVA22l*	156	18030.22	9.26	95.64	0.026	Chlo
	Spim04g032680.1.1	*SpiHVA22g*	323	35618.35	8.81	71.05	−0.346	Chlo
	Spim01g007550.1.1	*SpiHVA22a*	180	21270.52	5.95	92.11	−0.116	Cyto
	Spim09g019680.1.1	*SpiHVA22k*	241	28172.67	8.93	84.94	−0.026	Vacu
	Spim10g015050.1.1	*SpiHVA22m*	603	68493.8	8.68	81.51	−0.408	Nucl
	Spim04g027770.1.1	*SpiHVA22f*	494	56507.39	9.34	89.82	−0.226	Nucl
	Spim01g044830.1.1	*SpiHVA22b*	255	29350.56	8.68	108.94	0.308	Nucl
	Spim10g025730.1.1	*SpiHVA22n*	150	17597.28	9.1	80.73	−0.339	Cyto
*S.pennellii*	Sopen05g003250.1	*SpHVA22g*	171	20234.38	7.67	98.13	−0.102	E.R.
	Sopen11g005670.1	*SpHVA22m*	142	16848.74	8.58	95.42	0.063	Cyto
	Sopen03g028180.1	*SpHVA22b*	136	15845.88	9.37	115.44	0.21	Extr
	Sopen04g006560.1	*SpHVA22e*	131	15267.96	5.53	106.41	0.186	Extr
	Sopen03g030110.1	*SpHVA22c*	180	20364.57	6.65	104	0.076	Nucl
	Sopen06g029050.1	*SpHVA22h*	135	15652.57	8.8	104.74	0.231	Chlo
	Sopen10g022980.1	*SpHVA22k*	187	21851.19	7.02	83.9	−0.07	E.R.
	Sopen03g035280.1	*SpHVA22d*	176	20507.66	6.41	96.93	0.005	Extr
	Sopen10g003720.1	*SpHVA22i*	156	18020.24	9.24	93.14	0.013	Chlo
	Sopen12g031160.1	*SpHVA22n*	302	33844.67	9.5	68.15	−0.447	Chlo
	Sopen04g034960.1	*SpHVA22f*	304	33623.99	8.95	68.12	−0.379	Chlo
	Sopen10g032550.1	*SpHVA22l*	188	22108.6	8.63	86.7	−0.227	Extr
	Sopen10g018090.1	*SpHVA22j*	559	63615.22	8.69	81.82	−0.444	Nucl
	Sopen01g003320.1	*SpHVA22a*	155	18113.25	10.09	51.68	−0.977	Nucl
*S.lycopersicoides*	Solyd05g052440.1	*SlydHVA22h*	171	20176.3	7.67	97.6	−0.113	E.R.
	Solyd11g055150.1	*SlydHVA22n*	123	14697.04	7.9	91.14	−0.112	Cyto
	Solyd11g055100.1	*SlydHVA22m*	145	17473.82	8.63	123.66	0.675	Chlo
	Solyd03g072250.1	*SlydHVA22d*	190	21617.78	6.16	90.37	−0.049	Cyto
	Solyd06g073510.1	*SlydHVA22i*	135	15689.63	8.8	104.74	0.205	Chlo
	Solyd03g077950.1	*SlydHVA22e*	176	20507.66	6.41	96.93	0.005	Extr
	Solyd12g070530.1	*SlydHVA22o*	229	26296.16	9.05	65.15	−0.345	Chlo
	Solyd12g070590.1	*SlydHVA22p*	302	33831.62	9.28	68.15	−0.456	Chlo
	Solyd04g078410.1	*SlydHVA22g*	313	34770.38	8.87	68.02	−0.35	Chlo
	Solyd01g052910.1	*SlydHVA22a*	182	21641.02	5.91	95.88	−0.036	Nucl
	Solyd10g061800.1	*SlydHVA22l*	241	27758.68	7.05	78.92	−0.22	Chlo
	Solyd10g059860.1	*SlydHVA22k*	547	62120.36	8.28	79.85	−0.452	Nucl
	Solyd10g052770.1	*SlydHVA22j*	119	14080.39	9.14	85.21	−0.242	Chlo
	Solyd01g086740.1	*SlydHVA22b*	306	34201.23	8.87	110.56	0.206	Nucl
	Solyd03g070260.1	*SlydHVA23c*	88	9989.65	9.64	84.2	−0.172	Cyto

### Phylogenetic tree analysis of *HVA22* family genes

**Figure 1:**
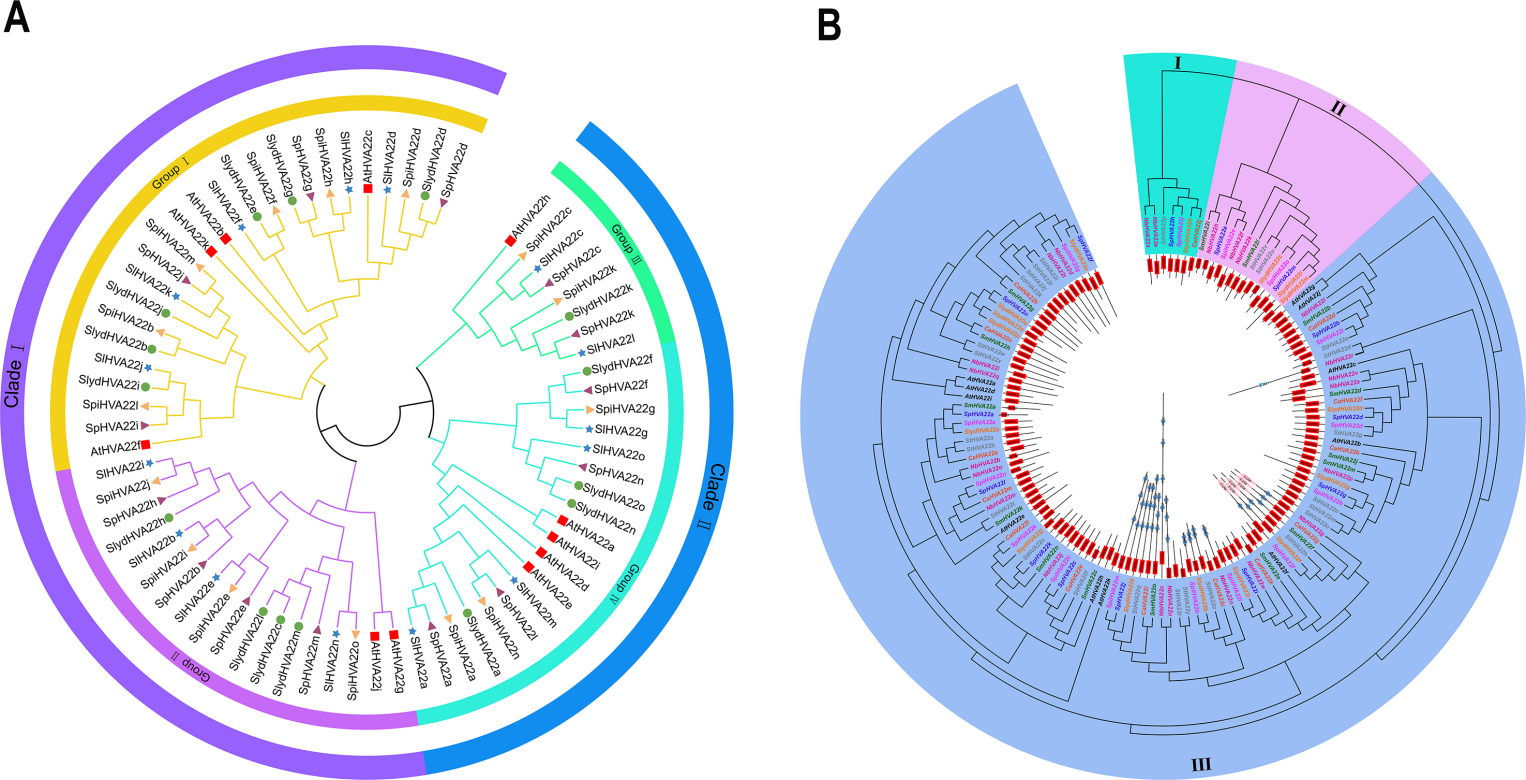
Phylogenetic tree analysis of the *HVA22* gene. (A) Phylogenetic trees were constructed for 60 *HVA22* genes form *Lycopersicon* using the ML method with 1,000 bootstrap repetitions. A species abbreviation was provided prior to each HVA22 protein name: Sl, *Solanum lycopersicum*; Sp, *Solaunm pennellii*; At, *Arabidopsis thaliana*; Spi, *Solaunm pimpinellifolium*; and Slyd, *Solaunm lycopersicoides*. (B) Phylogenetic tree of the *HVA22* family in Solanaceae. The phylogenetic tree was constructed using the ML method with 1,000 bootstrap repetitions. The different coloured *HVA22* genes were derived from different Solanaceae species, and the conserved structural domains of the corresponding *HVA22* genes are shown inside the evolutionary tree, with the TB2/DP1 structural domain in red, the Zf-met structural domain in blue, and the RVT-3 structural domain in pink.

**Figure 2:**
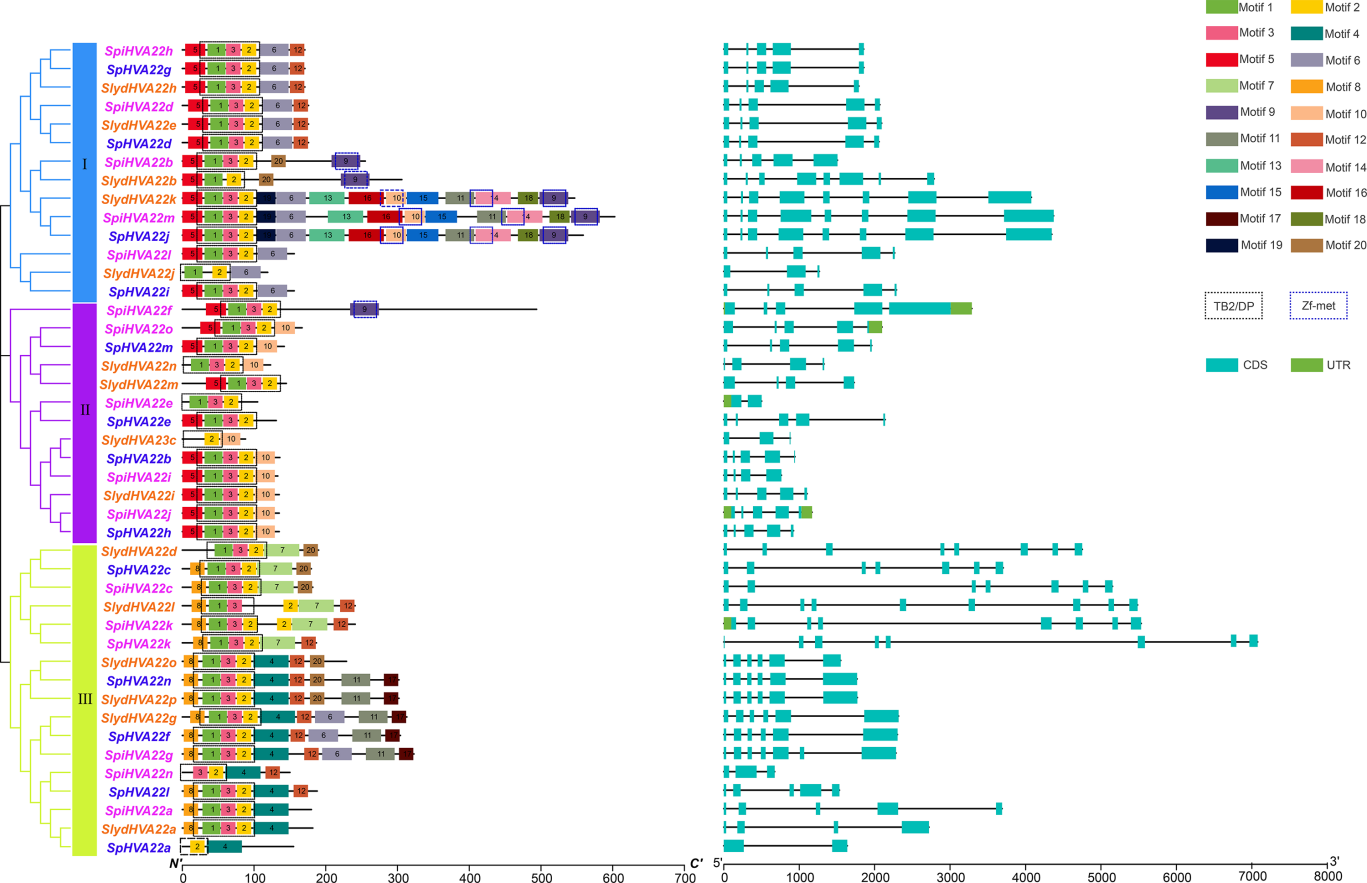
Genetic structure analysis. Phylogenetic relationships, Structure and conserved motifs of *HVA22* genes in three types of wild tomatoes. The different coloured *HVA22* genes were derived from different wild tomato species. Cyan boxes indicate exons, green boxes indicate UTR and black lines indicate introns. The numbers 1–20 and the different colored boxes indicate motifs.

We combined 45 *HVA22* genes from three species of wild tomatoes (16 in *S. lycopersicoides*, 14 in *S. pennellii*, and 15 in *S. pimpinellifolium*) with *HVA22* genes from *A. thaliana* and cultivated tomatoes (*S. lycopersicum*) to construct a complete phylogenetic tree (Table S3). The phylogenetic tree showed that *HVA22* genes in the cultivated tomatoes, three species of wild tomatoes, and *A. thaliana* were divided into two clades: I and II (Fig. 1A). The subgroups I and II were included in clade I, and the subgroups III and IV were included in clade II. The *HVA22* family genes in the three species of wild tomatoes in groups I, II, III, and IV clustered on a subgroup with the *A.thaliana* and cultivated *HVA22* genes. To enable a comprehensive understanding of the quantitative distribution of *HVA22* family gene members in tobacco (*N. benthamiana*), potato (*S. tuberosum*), eggplant (*S. melongena*), pepper (*C. annuum*), and three kinds of wild tomatoes (*S. pimpinellifolium, S. pennellii*, and *S. lycopersicoides*). We identified 120 *HVA22* family genes in selected Solanaceae species, constructed a phylogenetic tree, and designated these genes based on their chromosomal locations (Table S4). All *HVA22* genes were classified into three groups (Fig. 2B). Large differences were found in amino acid length and structural domains of *HVA22* family genes. HVA22 family proteins in group III had the most pronounced differences in length and structural domains. However, the amino acid lengths were essentially similar in each cluster in group III. The HVA22 family proteins in group III also possessed the Zf-met, RVT-3, and LRR_8 structural domains besides the TB2/DP structural domain unique to HVA22 proteins. In addition, the Solanaceous HVA22 family proteins with Zf-met (*SpiHVA22m, SpHVA22j, SlydHVA22k, StHVA22v, CaHVA22i, SmHVA22o, NbHVA22c, NbHVA22u, SpiHVA22b, SlydHVA22b, StHVA22 h*, and *NbHVA22n*) and RVT-3 (*SpiHVA22f, SlydHVA22f, StHVA22g, SmHVA22f, CaHVA22g,* and *NbHVA22g*) structural domains clustered in group III. On the contrary, *NbHVA22r* containing the LRR_8 structural domain was independently classified into a distinct cluster, which might be related to the fact that only *NbHVA22r* contained the LRR_8 structural domain among the numerous HVA22 family proteins in species belonging to Solanaceae. Moreover, the distribution position of the TB2/DP structural domain in the amino acid sequence of the HVA22 family in Solanaceae was at the N-terminal, except for *SlydHVA22m*, *NbHVA22a, CaHVA22l,* and *SmHVA22d*, which were at the C-terminal.

### Conserved motif and gene structure analyses of the *HVA22* gene family

The conserved motifs of the *HVA22* family genes of three wild tomato species were predicted using the online tool MEME to understand the specific distribution of conserved motifs in *HVA22* genes in the three species of wild tomatoes, and a total of 20 conserved motifs were identified (Fig. 2). Motifs 1, 2, and 3 formed the TB2/DP structural domain, which was distributed in *HVA22* family genes in the three species of wild tomatoes. Additionally, the Zf-met structural domain consisting of Motifs 9, 10, and 14 was also distributed in some wild tomato HVA22 family of proteins. The HVA22 protein possessing the Zf-met structural domain was mainly concentrated on a small cluster in group I. The analysis indicated that the amino acid motif composition of the HVA22 family in the same group was approximately similar. HVA22 genes containing a Zf-met structural domain exhibited more motifs, with the exception of *SpiHVA22f*, *SpiHVA22b*, and *SlydHVA22b*.

The structural differences in exon–intron arrangement are an important source of gene family variation and plant diversity. Different structures lead to differences in gene expression and function (Xu et al., 2012). Our results showed that the *HVA22* family genes in the three species of wild tomatoes were divided into three major groups by the phylogenetic tree, with a large degree of similarity in the exon–intron arrangement in most of the same clusters (Fig. 2). However, large differences existed in the arrangements in some of the clusters. In groups I and II, the number of exons was mostly 5, and the number of exons in a few individual *HVA22* family genes was 2 (*SpiHVA22e*), 3 (*SlydHVA22c*, and *SlydHVA22j*), 4 (*SlydHVA22n*, *SlydHVA22m*, and *SpiHVA22i*), and 8 (*SlydHVA22b, SlydHVA22k, SpiHVA22m,* and *SpHVA22j*). The number of group III exons was highly variable, ranging from 2 to 9. Despite the large variation in the number of exons in group III, the *HVA22* genes in each subgroup in group III exhibited similar gene structures.

### *HVA22* gene promoter analysis in the three species of wild tomatoes

**Figure 3:**
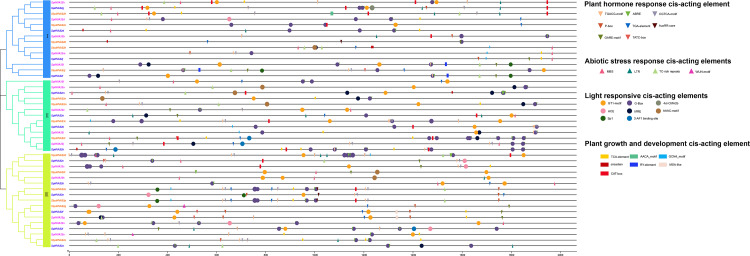
Distribution of CREs of *HVA22* genes in three species form wild tomato. Different colored squares indicate different branches of *HVA22* family genes in the phylogenetic tree. Different CREs were indicated by different shapes, inverted triangles indicate hormone response elements, circles indicate light response elements, boxes indicate growth and development related elements, triangles indicate stress response related elements, and different elements were indicated by different colors.

We performed a *cis-acting* element analysis of the 2,000-bp promoter sequence upstream of the *HVA22* gene in three species of wild tomatoes (*S. lycopersicoides*, *S. pennellii*, and *S. pimpinellifolium*) (Fig. 3). The analysis showed that the *cis*-acting elements in the *HVA22* gene were divided into four categories: light-responsive *cis*-acting elements, phytohormone-responsive *cis*-acting elements, biotic/abiotic stress *cis*-acting elements, and growth and development *cis*-acting elements. *Cis*-acting elements involved in light response, phytohormone response, and development were distributed in *HVA22* family genes in all three species of wild tomatoes, while *cis*-acting elements involved in plant growth and abiotic stress were only present in the promoters of some *HVA22* family genes. In the present study, five hormone response elements were identified to be involved in the transcriptional initiation of the *HVA22* gene: abscisic acid response element (ABRE), salicylic acid response element (TCA-element and SARE), gibberellin response element (TATC-box, GARE-motif and P-box), auxin-responsive element (TGA-element, AuxRR-core, and TGA-box), and methyl jasmonate response element (TGACG-motif and CGTCA-motif). Four response plant biotic/abiotic stress elements were found, namely, *cis*-acting elements involved in defense and stress response (TC-rich repeats, and WUN-motif), *cis*-acting elements involved in low-temperature response (LTR), and MYB-binding sites involved in drought-inducing elements (MBS). Six species (CAT-box, AACA_motif, GCN4-motif, circadian, RY-element, and MSA-like) were involved in plant growth and developmental response elements. The largest number of *cis*-acting element types were involved in light response, with eight light response elements identified in the *HVA22* family of genes; among these, except for *SpHVA22j*, the promoter sequences of the remaining *HVA22* family genes were distributed with *cis*-acting elements associated with light response. Among the promoters of the HVA22 family genes in the three species of wild tomatoes, apart from *SlydHVA22b, SlydHVA22c, SlydHVA22e, SlydHVA22f, SlydHVA22g, SlydHVA22j, SlydHVA22n, SlydHVA22o, SlydHVA22p, SpHVA22b, SpHVA22b, SpHVA22f, SpHVA22 h, SpHVA22i, SpHVA22n, SpiHVA22a, SpiHVA22g*, and *SpiHVA22l*, all other *HVA22* family genes contained *cis*-acting elements in response to low temperature or drought. Four *cis*-acting elements involved in biotic/abiotic stress were not as widely distributed in the promoters of *HVA22* family genes in the three species of wild tomatoes as were light-responsive elements and phytohormone-responsive elements. This also suggested that some *HVA22* family genes were involved in responding to abiotic stresses.

### Gene localization to chromosomes and collinearity analysis of *HVA22* in wild tomatoes

**Figure 4:**
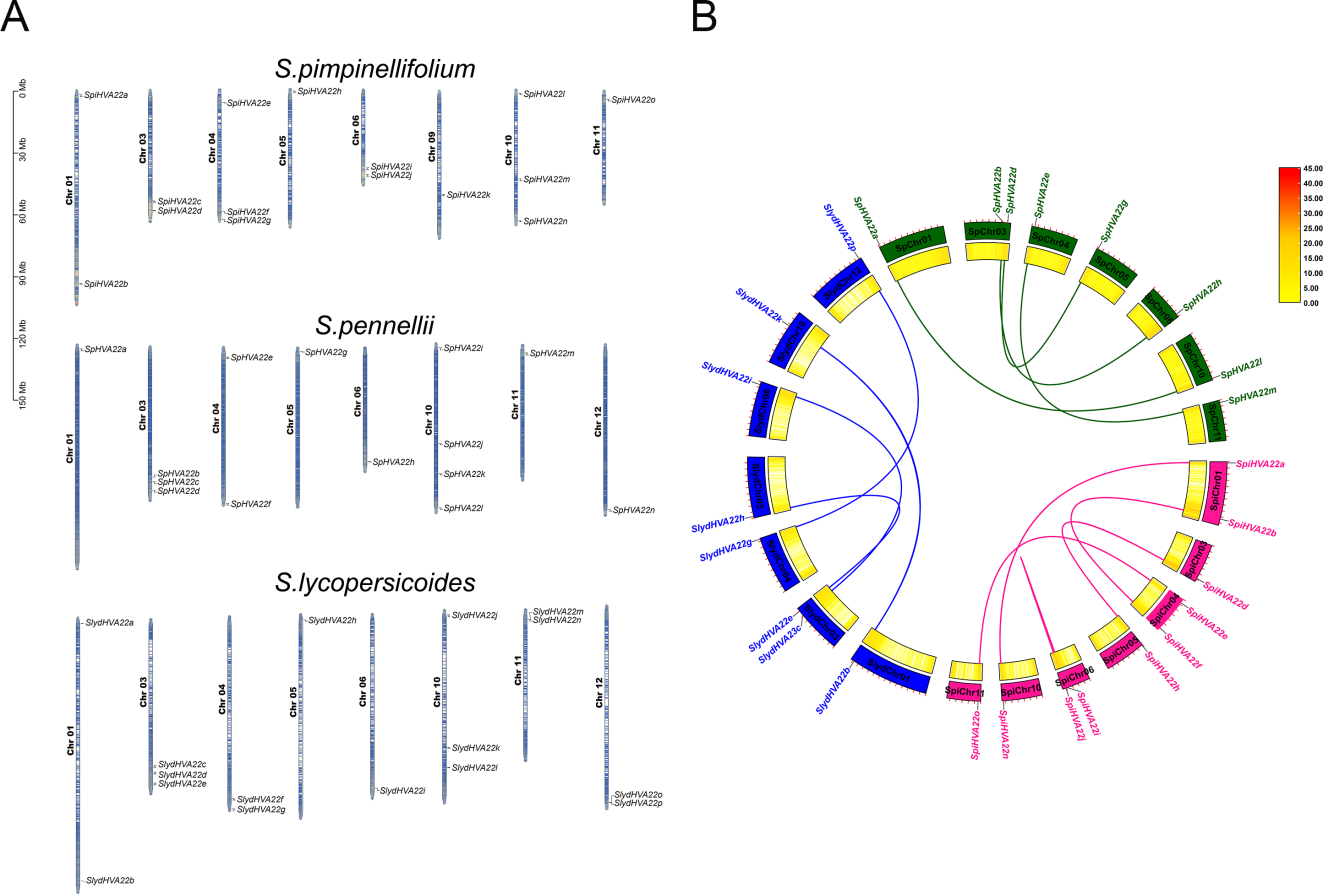
Gene localization to chromosomes and collinearity analysis of *HVA22* within species. (A) Chromosome localization of three species form wild tomato *HVA22* genes. (B) Collinearity analysis of *HVA22* within species. Pink, green, and blue lines, which indicate the colinearity of the HVA22 gene between *Solaunm pimpinellifolium* and *Solaunm pimpinellifolium*, *Solaunm pennellii* and *Solaunm pennellii*, and *Solaunm lycopersicoides* and *Solaunm lycopersicoides*, respectively.

The *HVA22* family genes in three species of wild tomatoes were mainly distributed on chromosomes Chr 01, Chr 03, Chr 04, Chr 05, Chr 06, Chr 09, Chr 10, Chr 11, and Chr 12 (Fig. 4A). Among these, the chromosomes distribution of the *HVA22* family genes in *S. pennellii* and *S. lycopersicoides* was consistent; they were all distributed on chromosomes Chr 01, Chr 03, Chr 04, Chr 05, Chr 06, Chr 10, Chr 11, and Chr 12. The chromosomal distribution of *HVA22* family genes in *S. pimpinellifolium* differed from that of *S. pennellii* and *S. lycopersicoides*. The *HVA22* family gene in *S. pimpinellifolium* was distributed on chromosome Chr 09; however, no *HAV22* gene family members were found on chromosome Chr 12. We performed intraspecific MCScanX analysis on three species of wild tomatoes to gain a clear understanding of the linear relationships between *HVA22* family genes within species. The results showed five pairs of paralogous homologous genes within the *HVA22* family in *S. pimpinellifolium*: *SpiHVA22o/SpiHVA22e*, *SpiHVA22n/SpiHVA22a*, *SpiHVA22j/SpiHVA22i*, *SpiHVA22h/SpiHVA22d*, and *SpiHVA22f/SpiHVA22b*. Four pairs of paralogous homologous genes existed in *S. lycopersicoides* and *S. pennellii* (*SlydHVA22b/SlydHVA22k*, *SlydHVA22c/SlydHVA22i*, *SlydHVA22e/SlydHVA22h*, and *SlydHVA22g/SlydHVA22p* in *S. lycopersicoides*; *SpHVA22a/SpHVA22l*, *SpHVA22b/SpHVA22n*, *SpHVA22d/SpHVA22g,* and *SpHVA22e/SpHVA22m* in *S. pennellii*) (Fig. 4B). We performed a Ka/Ks analysis of the identified paralogous homologs to enable a comprehensive understanding of the *HVA22* family genes in three species of tomatoes. The final results showed that all 13 pairs of paralogous homologous genes in the HVA22 families had Ka/Ks greater than 1 (Table 2). This also suggested that the paralogous homologous gene pairs were subjected to stronger environmental stresses, and the gene evolution and protein function were stabilized.

**Table 2:** The Ka/Ks ratios and date of duplication for duplicate HVA22 genes in three wild tomatos.

**Species**	**Duplicated gene pairs**	**Ka**	**Ks**	**Ka/Ks**	**Selective pressure**	**Type**
*S.lycopersicoides*	*SlydHVA2b/SlydHVA22k*	0.80688	1.83965	0.43861	Purify selection	Segmental
	*SlydHVA2c/SlydHVA22i*	0.43342	1.57164	0.27577	Purify selection	Segmental
	*SlydHVA22e/SlydHVA22h*	0.22811	3.48793	0.0654	Purify selection	Segmental
	*SlydHVA22g/SlydHVA22p*	0.18797	0.6627	0.28364	Purify selection	Segmental
*S.pimpinellifolium*	*SpiHVA22a/SpiHVA22n*	0.34191	2.79239	0.12244	Purify selection	Segmental
	*SpiHVA22b/SpiHVA22 f*	1.13033	3.13699	0.36032	Purify selection	Segmental
	*SpiHVA22d/SpiHVA22h*	0.24031	3.49311	0.0688	Purify selection	Segmental
	*SpiHVA22e/SpiHVA22o*	0.20661	0.78885	0.26192	Purify selection	Segmental
	*SpiHVA22i/SpiHVA22j*	0.17878	0.9293	0.19239	Purify selection	Segmental
*S.pennellii*	*SpHVA22a/SpHVA22l*	0.46107	3.2625	0.14132	Purify selection	Segmental
	*SpHVA22b/SpHVA22h*	0.18824	1.15914	0.16239	Purify selection	Segmental
	*SpHVA22d/SpHVA22g*	0.22977	3.50697	0.06552	Purify selection	Segmental
	*SpHVA22e/SpHVA22m*	0.18263	0.65296	0.27969	Purify selection	Segmental

### Evolution and collinearity analysis of the *HVA22* gene family in the three species of wild tomatoes

**Figure 5:**
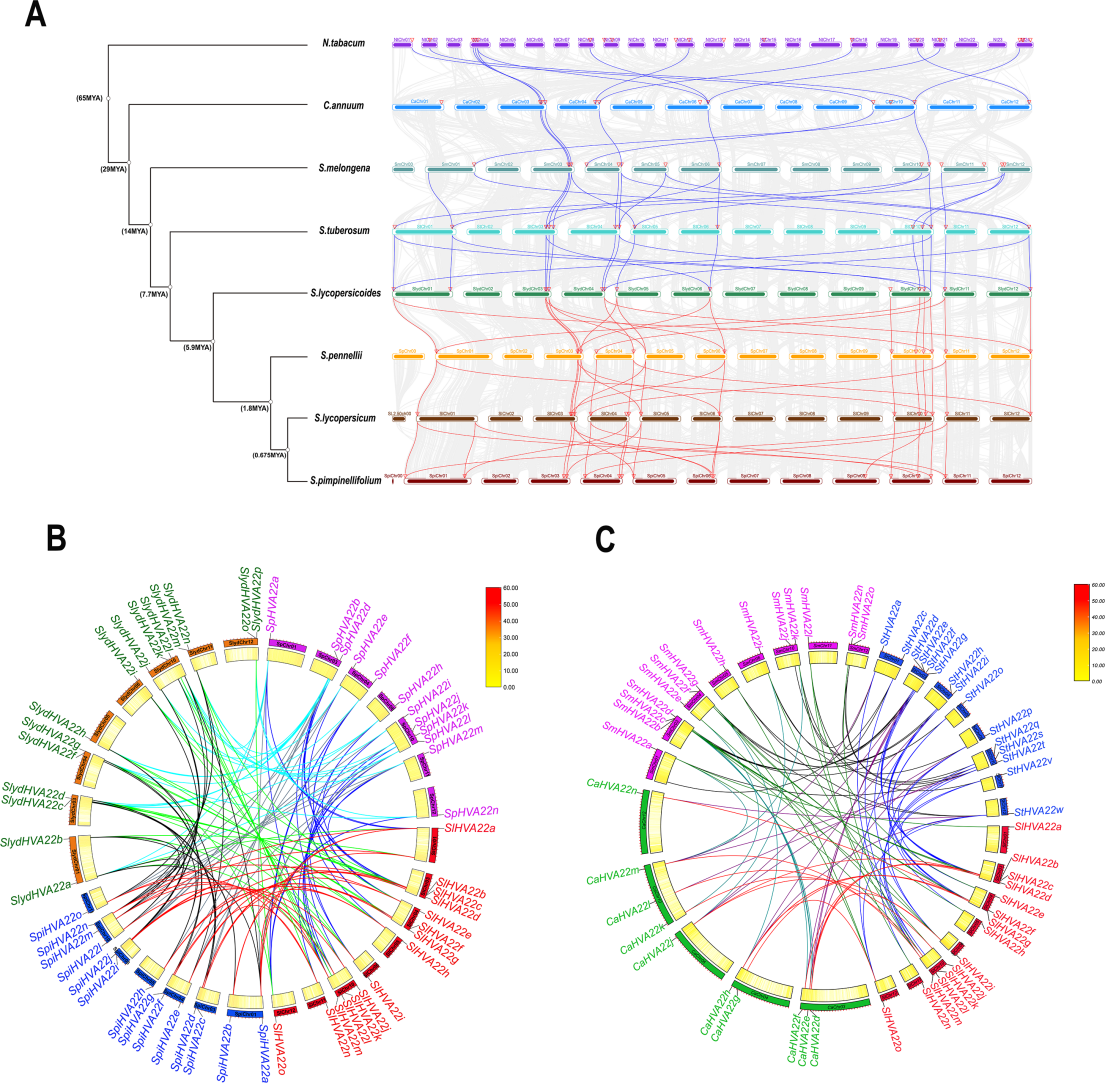
Homologous genes and evolutionary analysis of the *HVA22* family. (A) Co-lineage map for Solanaceae species, with species genomes arranged in evolutionary order and colored lines representing *HVA22* genes with direct homologous relationships within each species. (B) Collinearity of *HVA22* genes within *Solaunm pimpinellifolium*, *Solaunm lycopersicoides*, *Solaunm pennellii*, and *Solanum lycopersicum*, with the outer circle showing the chromosomes of each species, the inner circle showing the gene density, the two ends of the lines representing the direct homologous HVA22 genes, and the different colors indicate comparisons between different Lycopersicon. (C) Co-lineage map of *HVA22* genes within Solanaceae (*C. annuum*, *S. melongena*, *S. tuberosum*, and *S. lycopersicum*), with the outer circle showing the chromosomes of each Solanaceae, the inner circle showing gene density, the ends of the lines representing direct homologous *HVA22* genes, and the different colors indicate comparisons between different species.

We performed an interspecific collinearity analysis of *HVA22* family genes in eight Solanaceae species, tobacco, pepper, potato, eggplant, and four species of *Lycopersicon* (*S. lycopersicum*, *S. lycopersicoides*, *S. pennellii* and *S. pimpinellifolium*), based on their divergence times (Wu & Tanksley, 2010), to explore the homology of *HVA22* family genes in Solanaceae plants. Our results showed a significant increase in *HVA22* family homologous genes and a significant acceleration in the rate of evolution from pepper onward. The distribution of *HVA22* homologs was similar on the chromosomes of the remaining Solanaceae members except for tobacco. The *HVA22* genes on chromosomes Chr 03, Chr 04, Chr06, and Chr10 in Solanaceae (excluding tobacco) had high homology. A large similarity and homology were seen in the chromosomal distribution of *HVA22* genes in four species of *Lycopersicon* (Fig. 5A). To gain further insight into the homology of *HVA22* family genes in Solanaceae, we performed a collinearity analysis of *HVA22* family genes in Solanaceae plants one by one; the analysis was also performed in the four species of *Lycopersicon* by the same method. The final results showed that most of the *HVA22* family genes in the three species wild tomatoes were orthologous to each other and the cultivated tomato *HVA22* family genes. Among these, the cultivated tomato *HVA22* genes were found to have corresponding homologs in all three species of wild tomatoes, except *SlHVA22o*, which was not identified as a direct homolog in *S. piminellifolium* (Fig. 5B). The distribution of the *HAV22* family genes on the chromosomes of the four tomato species was also highly similar. The *HVA22* genes on chromosome Chr03 were found to be highly homologous to each other in the co-linearity analysis of Solanaceae (tomato, pepper, potato, and eggplant). However, the *SlHVA22a* and *SlHVA22e* genes located on chromosomes SlChr01 and SlChr04 were found to be orthologous only in eggplant (*SmHVA22a* and *SmHVA22l*), while no orthologous genes were found in pepper and potato. The orthologous genes of *SlHVA22e*, *SlHVA22h*, and *SlHVA22n* located on chromosomes SlChr04, SlChr05, and SlChr11 were present in potato (*StHVA22v, StHVA22o*, and *StHVA22v*) and eggplant (*SmHVA22l*, *SmHVA22c*, and *SmHVA22l*); their orthologous genes were not detected in pepper (Fig. 5C).

### Protein–protein network analysis of *HVA22* family genes in tomato

**Figure 6:**
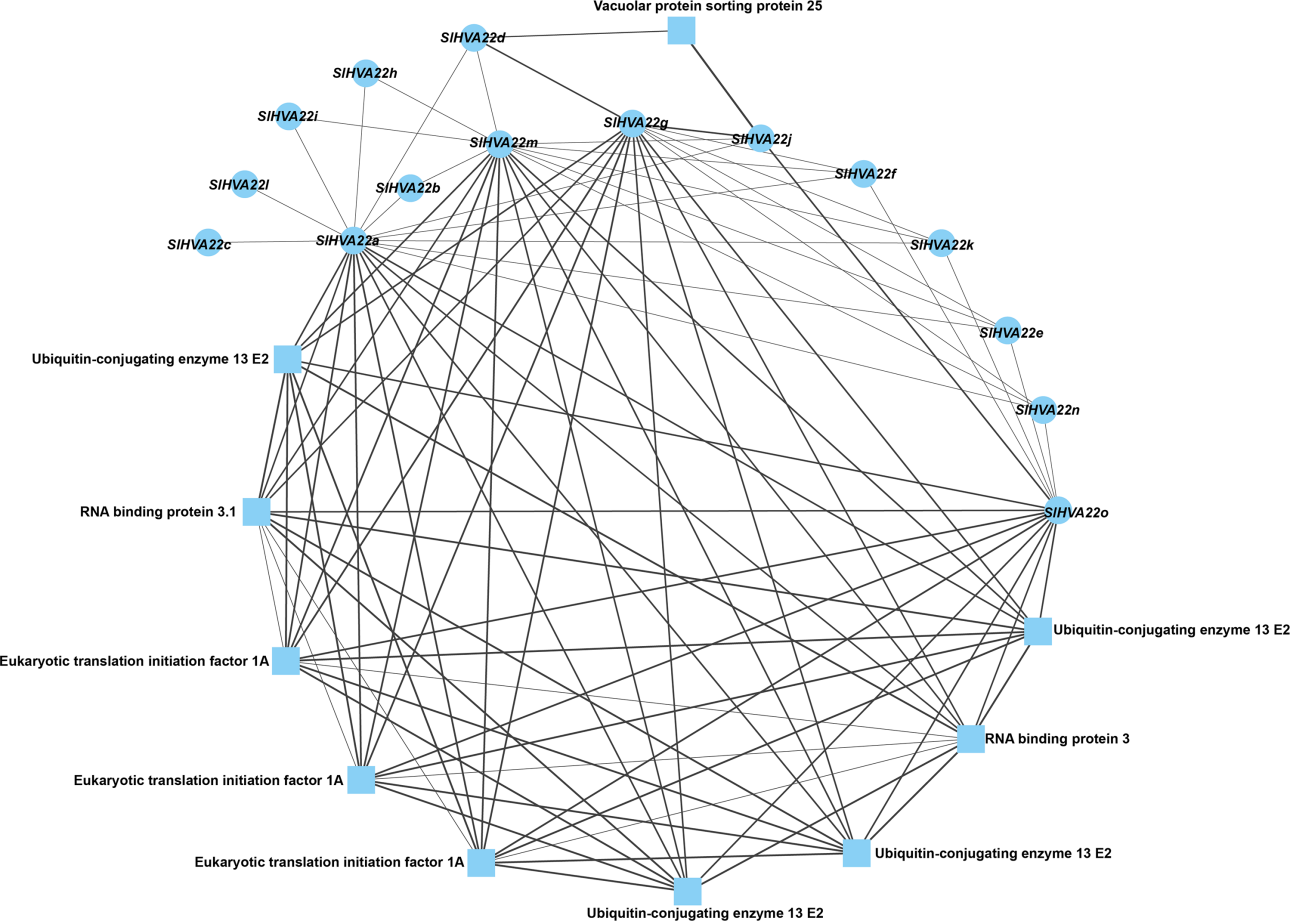
Interaction network of the HVA22 family with other proteins. Each node represents a protein, each connecting line represents the presence of an interaction, and the thickness of the line represents the value of the composite score, with circles representing HVA22 proteins and squares representing other proteins.

We constructed a protein–protein network expression profile of tomato HVA22 family genes using the STRING database to investigate the interactions between HVA22-like proteins and other proteins. Our results showed interactions between *HVA22* family member proteins in tomatoes. Some *HVA22* family proteins (*SlHVA22o*, *SlHVA22g*, *SlHVA22m*, and *SlHVA22a*) interacted with ubiquitin-conjugating enzyme (ubiquitin-conjugating enzyme 13 E2), RNA-binding protein (RNA-binding protein 3.1), and eukaryotic translation initiation factor (ETA). Among these, *SlHVA22j* and *SlHVA22d* interacted with the vesicle-sorting protein (vacuolar protein sorting protein 25). The remaining *HVA22* family proteins (*SlHVA22f*, *SlHVA22k*, *SlHVA22e*, *SlHVA22n*, *SlHVA22b*, *SlHVA22i*, *SlHVA22h*, *SlHVA22l*, and *SlHVA22c*) interacted with proteins from their own family; interactions between them and with other proteins were not observed (Fig. 6).

### Expression of tomato *HVA22* gene in different tissues

**Figure 7:**
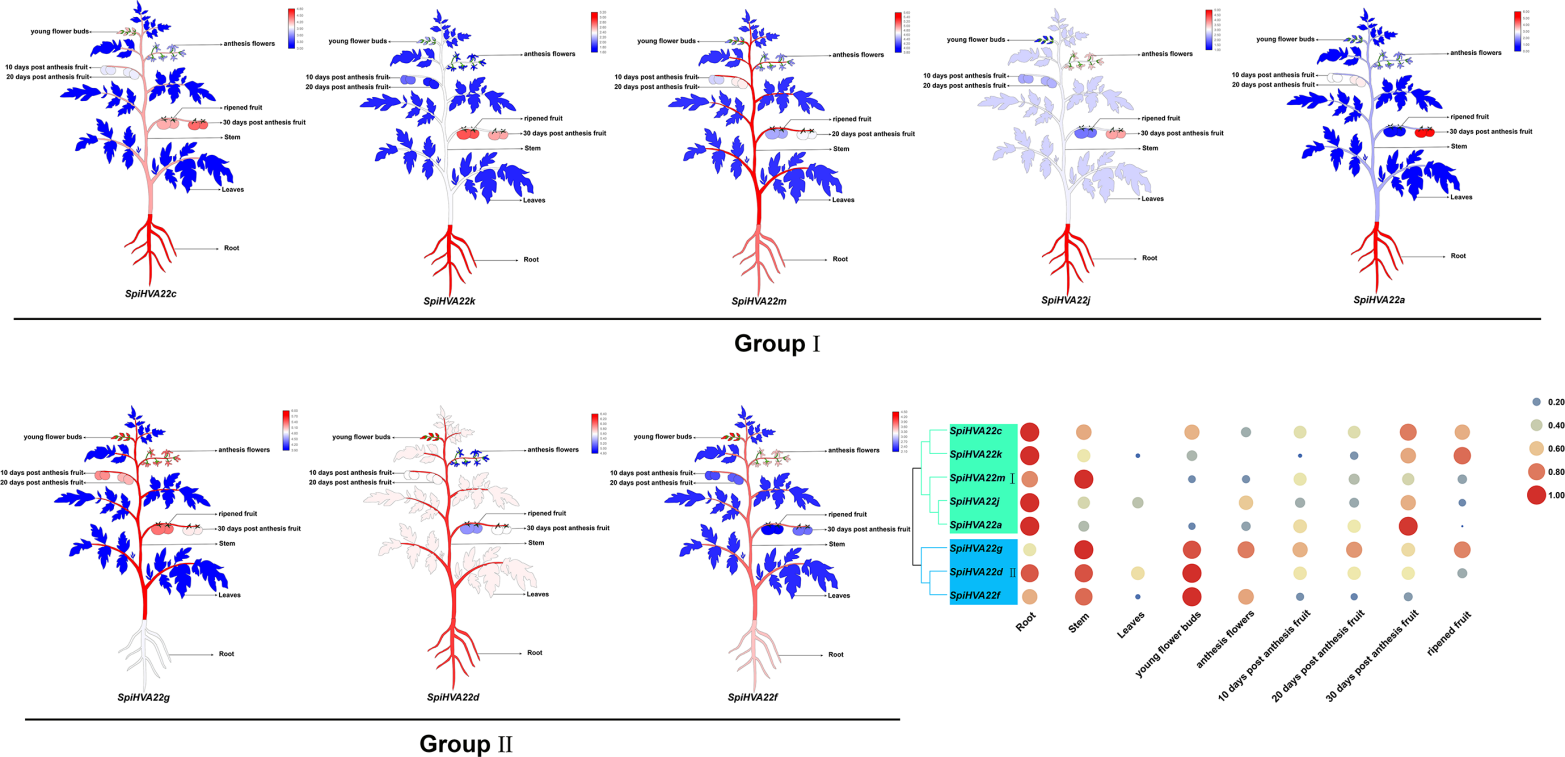
Heat map of tissue-specific expression of *HVA22* genes in *Solaunm pimpinellifolium*. Root, stem, leaves, young flower buds, anthesis flowers, 10 days post anthesis fruit, 20 days post anthesis fruit, 30 days post anthesis fruit, ripened fruit.

**Figure 8:**
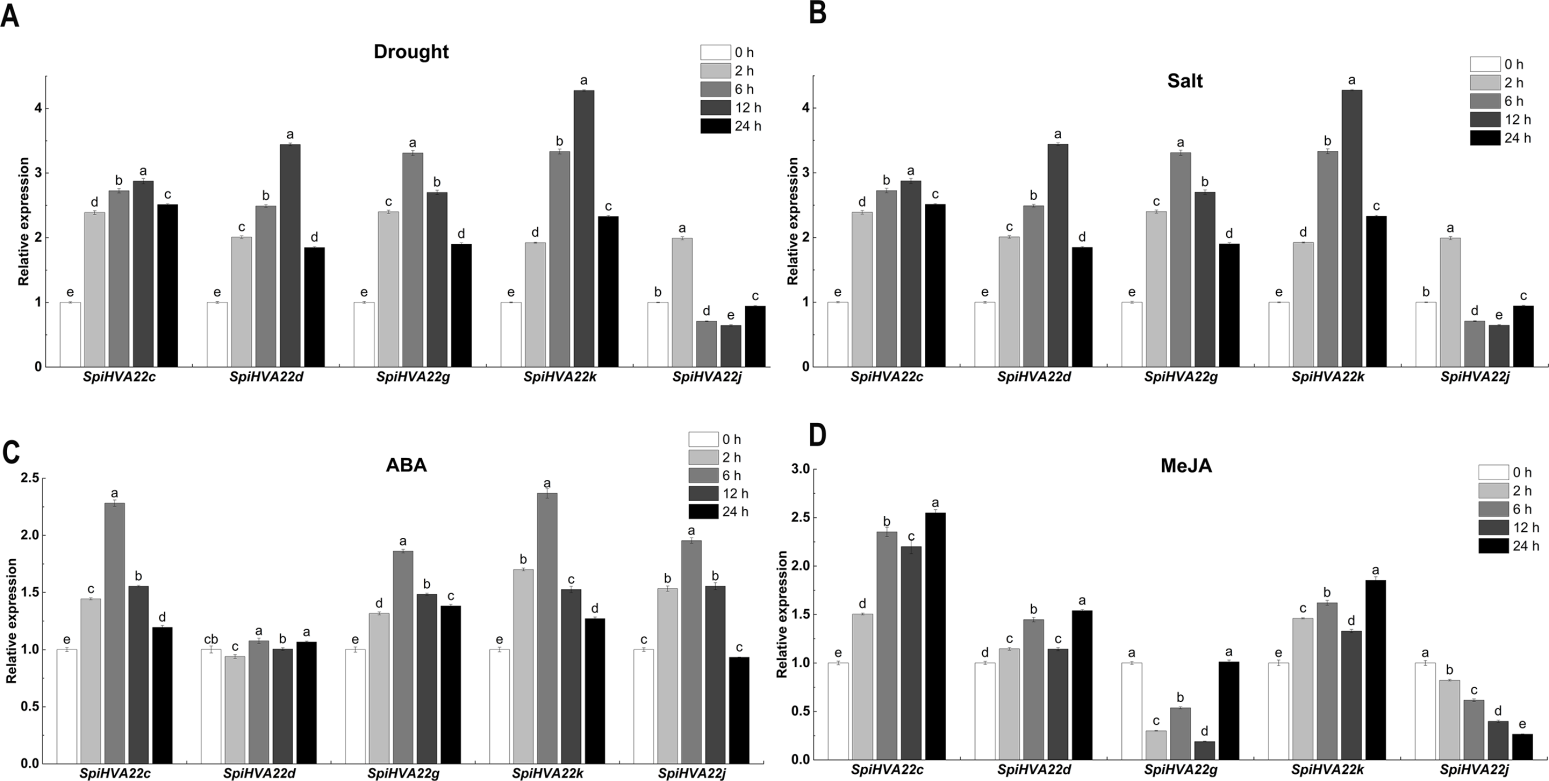
QRT-PCR validation of the *Solaunm pimpinellifolium HVA22* genes under abiotic stress and hormone induction. The standard deviations are shown with error bars.

We used published RNA-seq data to map the gene expression heat map of the *HVA22* gene in different tissues of *S. pimpinellifolium* to better understand the role of the *HVA22* gene in the growth and development of wild tomatoes. The results showed that most of the *HVA22* family members in wild tomatoes were expressed at a lower level in leaves and fruits. In particular, *SpiHVA22c*, *SpiHVA22k*, *SpiHVA22a*, and *SpiHVA22g* family member genes were highly expressed in ripening fruits. In addition, among the wild tomato *HVA22* family genes, *SpiHVA22d* was slightly more expressed in leaves compared with other *HVA22* family members. The roots showed the highest expression of the remaining *HVA22* family members, with the exception of *SpiHVA22g*. The expression of *HVA22* family genes in group I was generally low in flower buds. However, the *HVA22* family member genes in group II showed higher expression levels in the flower buds. In addition, the *HVA22* member genes in group II also showed higher expression levels in the stems. Overall, the expression of tomato *HVA22* family genes was concentrated in roots, flowers, and developing fruits (Fig. 7). The high expression of some *HVA22* family genes in roots also confirmed the possibility that *HVA22* family genes were involved in abiotic stress processes in tomatoes. This result provided important clues for our study of the function of the *HVA22* family gene in tomatoes. We treated *S. pimpinellifolium* seedlings with NaCl, PEG6000, and hormones (ABA and MeJA) to be more confident about how well the wild tomato *HVA22* family genes could respond to salt, drought, and hormones. The expression of *SpiHVA22c, SpiHVA22d*, *SpiHVA22g*, *SpiHVA22k*, and *SpiHVA22j* genes in the leaves of the plants obtained from the treatments was analyzed using qRT-PCR. Our results showed that the expression of *SpiHVA22c*, *SpiHVA22d*, *SpiHVA22g*, and *SpiHVA22k* was significantly higher under NaCl and PEG6000 treatment than in the control group. The expression of the *SpiHVA22j* gene was the highest after 2-h treatment and showed lower expression than that in the control group as the treatment time was extended. At the same time, the expression of *SpiHVA22k, SpiHVA22g,* and *SpiHVA22c* genes under ABA and MeJA treatment was similar to that under PEG6000 and NaCl treatment. Intriguingly, the *SpiHVA22g* and *SpiHVA22j* gene showed negative regulation under MeJA treatment and positive regulation under ABA treatment (Fig. 8). Apart from this, no significant change was found in the expression of the *SpiHVA22d* gene under ABA treatment, indicating that the *SpiHVA22d* gene was not regulated by the hormone ABA.

## Discussion

With advances in gene sequencing technology, next-generation sequencing has improved the accuracy of the genome, thereby avoiding annotation errors in individual gene sequences by whole-gene sequencing. This has also facilitated genetic improvement and directed breeding in tomatoes (Rothan, Diouf & Causse, 2019). The *HVA22* gene is commonly found in eukaryotes (Lu, 2013). It is expressed in plants in different tissues such as seeds, stems, and roots and is induced under several environmental stress conditions (*e.g.*, cold, salt, and drought) mediated by ABA (Shen et al., 2001). The *HVA22* gene family has been reported in *A. thaliana* (Chen et al., 2002), *C. clementina* (Gomes Ferreira et al., 2019), and cultivated tomatoes (Wai et al., 2022), but has not been reported in wild tomatoes (*S. lycopersicoides*, *S. pennellii,* and *S. pimpinellifolium*). The results of the phylogenetic tree showed that the three wild tomato *HVA22* family genes were divided into two clades, which were further divided into four groups (Fig. 1A). This result was also consistent with the classification of tomato *HVA22* family genes (Wai et al., 2022). Our results showed that the three wild tomato *HVA22* family genes in each group were well assigned to the known HVA22 groups of *A. thaliana* and cultivated tomatoes. This further demonstrated the highly conserved nature of the *HVA22* family of genes in *Lycopersicon* crops. We constructed a phylogenetic tree using *HVA22* family genes from seven species of Solanaceae to characterize the *HVA22* family genes more comprehensively. The results showed that the *HVA22* family genes in seven species of Solanaceae were divided into three major branches (Fig. 1B). Although the number of *HVA22* family members varies significantly among Solanaceae, they all carry the TB2/DP1 structural domain specific to HVA22-like. In addition, some of the HVA22 proteins have other structural domains besides TB2/DP1, and HVA22 proteins with other structural domains are clustered together in a phylogenetic tree. This also illustrates the reliability of the phylogenetic structure and the high homology of the *HVA22* gene in Solanaceae. This homology may result from the TB2/DP1 structural domain specific to the HVA22 protein. In addition, the analysis of the physicochemical properties of HVA22 family proteins in the three species of wild tomatoes found that the average number of amino acids was 231 (*S. pimpinellifolium*), 208 (*S. pennellii*), and 217 (*S. lycopersicoides*) (Table 1). These were close to the 230 amino acids in tomato HVA22 (Wai et al., 2022), but showed a large difference from the 130 amino acids of barley (Shen et al., 2001) and 151 amino acids of *C. clementina* (Gomes Ferreira et al., 2019), which might be related to the large evolutionary distance between different species. Interestingly, we found that the chromosomal distribution positions of the *HVA22* family genes in the three species of wild tomatoes showed striking similarities (Fig. 4A). At the same time, these *HVA22* proteins with similar distributions on the chromosome showed nearly identical physicochemical properties (*e.g.*, theoretical pI, molecular weight, aliphatic index, and grand average of hydropathicity) (Table 1). This also fully corroborated the conserved nature of the *HVA22* family genes in tomatoes.

The prediction of subcellular localization of HVA22 helps us understand the function of this gene. Our findings regarding the subcellular localization of *HVA22* genes in the three species of wild tomatoes revealed that a majority of the *HVA22* genes were predicted to be localized in the endoplasmic reticulum, chloroplast, cytoplasm, and nucleus (Table 1). Evidence showed that after heterologous expression, tomato SlHVA22a, SlHVA22f, and SlHVA22n proteins in rice protoplasts were observed to be localized to the endoplasmic reticulum (Wai et al., 2022). In addition, the rice *HVA22* family gene *OsHLP1* promoted the mechanism of disease resistance by compromising endoplasmic reticulum homeostasis when plants were infected by pathogens (Meng et al., 2022). In yeast, the *HVA22* gene homolog Yop1p played an important role in transporting material between the endoplasmic reticulum and the Golgi apparatus (De Antoni et al., 2002). These findings consistently showed that HVA22 played an important role as an endoplasmic reticulum localization protein in the life activities of eukaryotic cells. The HVA22 protein in the three species of wild tomatoes also seemed to exhibit similar characteristics.

The diversity of exon–intron structures is one of the key factors in the evolution of gene families and underpins the structure of phylogenetic trees (Shiu & Bleecker, 2003; Wang et al., 2014). The structural analysis of the *HVA22* family of genes in the three species of wild tomatoes showed that the number of exons was similar in groups I and II; however, a large difference was found in the number of exons in group III (Fig. 2). This also implied that the *HVA22* gene in the three species of wild tomatoes could be transcriptionally diversified through processes such as selective splicing to regulate a more complex and broad range of functions. A total of 20 motifs were identified in the structural analysis of the *HVA22* family genes in wild tomatoes, among which Motifs 1, 2, and 3 are important motifs that made up the TB2/DP1 structural domain of the *HVA22* gene. A membrane protein TB2-like1 exists in animals and belongs to the same TB2/DP1/HVA22 family of proteins. This protein may play an important role in the cell membrane transport of retinal ganglion cells (Sato et al., 2005). The Yop1p gene in yeast (*Saccharomyces cerevisiae*) is homologous to the barley *HVA22* gene, and the proteins it encodes have the TB2/DP2 structural domain (Brands & Ho, 2002). Previous studies showed that yeast Yop1p was an integral membrane protein with a hydrophilic region at its N-terminal end. This region bound specifically to the yeast Yip1p protein, and Yop1p interacted with Yip1p to mediate the biological process of intracellular membrane transport (Calero, Whittaker & Collins, 2001). Also, this membrane protein played a critical physiological role in barley, yeast, and humans (Sato et al., 2005; Voeltz et al., 2006). In a study of the TB2/DP1 structural domain of the *HVA22* protein of barley dextrin, the TM1, TM2, and TM3 segments of the *HVA22* gene were separately deleted using truncating mutations, so that the separately deleted *HVA22* genes would all carry their green fluorescent protein (GFP) tags, which were transferred to barley dextrin cells for cellular sublocalization observation. It was found that the sublocalization of the deletion of TM2 presented a results significant differences compared with *HVA22*:: GFP (Guo & David Ho, 2008). It further indicated that *HVA22* had the properties of a membrane protein. It also showed that the barley *HVA22* protein had a transmembrane region in the TB2/DP1 structural domain, and the presence of this transmembrane region provided a theoretical basis for the localization of the *HVA22* protein to the organelle membrane. This result was similar to our predicted subcellular localization of the *HVA22* gene in the three species of wild tomatoes. This also suggested that the *HVA22* gene with the TB2/DP1 structural domain might play an important role in intracellular vesicle transport (Brands & Ho, 2002). However, no relevant studies have been reported on the involvement of the HVA22 gene in intracellular vesicle transport in tomatoes. Moreover, the Zf-met structural domain was found in the amino acid sequence of HVA22 in the three species of wild tomatoes. The Zf-met domain is another zinc-finger domain containing the CxxC(12)Hx(6)H motif, which is associated with RNA binding (Yadav, Fernández-Baca & Cannon, 2020). This is similar to the structure identified in the tomato HVA22 protein. This further indicates that HVA22 family members are conserved in *Lycopersicon*.

*Cis*-acting elements are noncoding DNA sequences present in the promoter region of a gene. The distribution of different types of *cis*-acting elements in the promoter region may determine gene regulation and functional roles (Hernandez-Garcia & Finer, 2014). In this study, we characterized a 2,000-bp promoter sequence upstream of the *HVA22* family genes in the three species of wild tomatoes (Fig. 3). The *cis*-acting elements of *HVA22* family genes in the three species of wild tomatoes were divided into four main categories: *cis*-acting elements involved in light response, *cis*-acting elements involved in phytohormone response, *cis*-acting elements involved in biotic/abiotic stress, and *cis*-acting elements involved in growth and development. Common *cis-acting* elements associated with light response were ACE, MRE, G-box, GT1-Mofit, Sp1, 4cl-CMA2b, 3-AF1-binding site, and AAAC-Motif. These light-responsive *cis*-acting elements played an important regulatory role in stress response and growth and development of plants (Kaur et al., 2017). The *cis*-acting elements responding to phytohormones in the promoter of the *HVA22* gene family of three species of wild tomatoes were more widely distributed, including gibberellin, ABA, methyl jasmonate, ethylene, and salicylic acid. This further suggested that the HVA22 gene might be involved in the life activities of tomatoes through a hormone-regulated network, which was consistent with the findings in tomatoes and *C. clementina* (Gomes Ferreira et al., 2019; Wai et al., 2022). In addition, *cis*-acting elements involved in multiple stresses were predicted in the *HVA22* promoter in the three species of wild tomatoes, such as MBS (drought-inducible), LTR (low temperature responsive), and TC-rich repeats (defense and stress responsive). The *HVA22* gene response to low temperature and drought stress has been reported in *A. thaliana* (Chen et al., 2002), barley (Shen et al., 2001), and rice (Zhao et al., 2021). Six *cis*-acting elements associated with growth and development were identified in the promoters of *HVA22* family genes in the three species of wild tomatoes, including RY-element (involved in seed-specific regulatory element), MSA-like (involved in cell cycle regulatory element), GAT-box (involved in meristematic tissue expression element), circadian (involved in circadian control regulatory element), AACA_motif (involved in the endosperm-specific negative expression), and GCN4_motif (involved in endosperm expression element) (Zhang et al., 2013). The *HVA22* gene promoter contained *cis*-acting elements associated with plant development, particularly involved in seed-specific regulatory elements, which might be relevant to the function of the *HVA22* gene in seed maturation and dormancy in barley (Guo & David Ho, 2008). However, further experimental evidence is needed by cloning the upstream promoter of the *HVA22* gene in the three species of wild tomatoes to obtain the corresponding experimental evidence and provide direction for the next study of the *HVA22* gene function.

Gene duplication provides the material basis for plant evolution and the generation of new functions (Huang et al., 2022). It occurs in several different modes, such as whole-genome duplication, single-gene duplication, and segmental duplication (De Bodt, Maere & Van de Peer, 2005; Paterson et al., 2010). The chromosomal localization and collinearity analysis of the *HVA22* gene in the three species of wild tomatoes showed spacer regions in the physical location of the *HVA22*-encoding gene on the chromosome in the three species of wild tomatoes (Fig. 4A). Thus, it was tentatively determined that the *HVA22* gene was amplified in the three species of wild tomatoes mainly by large-scale segmental duplication or whole-genome duplication of the gene family. Subsequently, the intraspecific collinearity analysis of the *HVA22* family genes of the three species of wild tomato showed that the *HVA22* genes were amplified in the three species of wild tomatoes in an all-segmental duplication manner, which was consistent with our previous speculation on the distribution of *HVA22* genes on chromosomes (Fig. 4B). At the same time, the same results were obtained in cultivated tomatoes (Wai et al., 2022). The ratio of nonsynonymous to synonymous substitutions reflects, to some extent, the selective pressure of gene evolution. Ka/Ks >1 represents positive selection for accelerated evolution, and Ka/Ks <1 represents the presence of purifying selection for gene duplication (Wang et al., 2010). The *HVA22* paralogous homologous gene pairs in all three wild tomatoes had Ka/Ks <1, which further suggests that these homologous gene pairs underwent more intense environmental selection pressure and exhibited functional homogeneity during evolution. Genome-wide duplication events (WGD) have long been recognized as an important evolutionary force in species formation, adaptation to the environment, and shaping of species diversity (Wood et al., 2009; Soltis & Soltis, 2016). Dicotyledons experienced *γ* events in gene duplication; however, Solanaceae members experienced another WGD event (T event) about 65 million years ago (Knapp, 2012; Wu, Han & Jiao, 2020). *HVA22* genes were added or lost in Solanaceae driven by genome-wide duplication events. As the Solanaceae diverged during evolution, the *HVA22* family gene members showed a decreasing trend and then an increasing trend in Solanaceae (Fig. 5A). This might be related to the contraction and expansion of family genes in Solanaceae when they experienced the most recent T event. The *HVA22* gene on SlChr 03 in tomatoes is the most conserved member of the Solanaceae family (Fig. 5B). For *HVA22* family genes present only in tomatoes but not in other members of Solanaceae, this might be due to gene deletions caused by genome-wide duplication events (Fig. 5C). The result that *HVA22* family genes were highly conserved in the four species of *Lycopersicon* also indicated the importance of *HVA22* genes in the life activities of tomatoes.

The ubiquitin–proteasome system (UPS) regulates various biological functions in plants, such as hormonal responses (Chen et al., 2018; He et al., 2018), abiotic stress responses (Kim, Jang & Seo, 2016; Shu & Yang, 2017), plant growth and development (Cho et al., 2011; Koops et al., 2011), circadian rhythms (Gil et al., 2017), and plant immune responses (Lin et al., 2008; Luo et al., 2010). The predicted results of tomato *HVA22* protein interactions indicated that the *SlHVA22* protein was involved in plant signaling and the regulation of plant growth and development (Fig. 6). Among these, the tomato *HVA22* protein was co-expressed with ubiquitin-binding enzymes, suggesting that the tomato *HVA22* gene might be involved in the ubiquitination and regulation of tomato growth and development and abiotic stresses. In addition, the vesicle sorting protein (VPS) (Xiang, Etxeberria & Ende, 2013) plays an important role in the protein sorting pathway as an important protein for the formation of endosomal sorting complex protein (ESCRT) (Gao et al., 2017). Previous studies showed that the growth hormone transporter proteins PIN1, PIN2, and AUX1 were the cargo proteins of ESCRT. To some extent, this suggested that VPS played a role in hormone signaling (Spitzer et al., 2009). The results of the co-expression of *HVA22* protein with VPS suggested that the *HVA22* protein might be a cargo membrane protein of ESCRT and played an important role in intracellular vesicle transport. RNA-binding proteins interacted with RNA through the RNA-binding domain to regulate RNA metabolism and function. Conversely, RNA could bind to RAN-binding proteins and affect their lifespan and function (Hentze et al., 2018). The co-expression of RNA-binding proteins with the tomato *HVA22* protein might indicate an important contribution of RNA-binding proteins in maintaining the function and longevity of the *HVA22* gene. The results of co-expression of the tomato *HVA22* gene with eukaryotic translation initiation factor 1A also further confirmed the conclusion that the HVA22 gene was present only in eukaryotes. Recently, that the AtGCN2 activation of eukaryotic translation initiation factor 2 phosphorylation was shown to be another key component in response to endoplasmic reticulum stress in *A. thaliana*, and it played an important role in the signaling process of the unfolded protein response (Afrin et al., 2020; Howell, 2021; Liu, Afrin & Pajerowska-Mukhtar, 2019).

The *HVA22* gene was differentially expressed in different tissues of *S. pimpinellifolium*, while the expression of individual *HVA22* genes showed an increasing trend during *S. pimpinellifolium* fruit development (Fig. 7). The root system, as the main tissue directly sensing drought and salt ions, has a high sensitivity to drought and salt ions. The high expression of the HVA22 gene in roots indicated that the HVA22 gene played an important role in plant root development and resistance to abiotic stress response. The expression of *HVA22* family genes showed an increasing trend as the fruit grew during fruit development. The same result was shown in the HVA22 family genes in cultivated tomatoes (Wai et al., 2022). This might be related to the involvement of *HVA22* family genes in tomato fruit growth and development. We treated *S. pimpinellifolium* seedlings with NaCl, PEG6000, and hormones, thereby performing qRT-PCR analysis of the *HVA22* family genes in *S. pimpinellifolium* (Fig. 8). The results showed that *SpiHVA22d*, *SpiHVA22g*, *SpiHVA22k*, and *SpiHVA22c* genes responded positively to salt and drought stresses. This was also similar to the characterization of *HVA22s* described in barley, *A. thaliana*, *C. clementina*, and cultivated tomatoes (Wai et al., 2022). In addition, the responses of *SpiHVA22k*, *SpiHVA22c*, and *SpiHVA22d* genes to ABA and MeJA also suggested that *SpiHVA22k*, *SpiHVA22c*, and *SpiHVA22d* genes played important roles in the ABA and MeJA pathways. Interestingly, the *SpiHVA22j* gene in the *S. pimpinellifolium* HVA22 family of genes showed negative regulation under MeJA treatment. This might be related to the fact that the *SpiHVA22j* gene was not involved in the plant jasmonic acid pathway. Previous studies in barley also pointed to the role of the *HVA22* gene as an early ABA-inducible gene (Shen et al., 2001). Our qRT-PCR results further concluded that the *S. pimpinellifolium HVA22* gene might be involved in the plant ABA pathway, which in turn responded to plant regulation of abiotic stresses.

## Conclusions

In the present study, we systematically identified *HVA22* family genes in tomatoes. We used a bioinformatics approach to describe the physicochemical properties, gene structure, *cis*-acting elements, and protein interactions of different *HVA22* genes. The expansion and contraction of *HVA22* family genes during the evolution of Solanaceae species were also discussed. The expression profile data of different tissues of *HVA22* family genes in tomatoes showed that the expression of *HVA22* family genes was mainly concentrated in roots, flowers, and developing fruit. We validated five genes in the tomato *HVA22* family using qRT-PCR. Four of these genes were involved in ABA- and MeJA-mediated regulatory pathways, and played important roles in tomato resistance to abiotic stresses (salt and drought). These results laid the foundation for further investigation of the function of *HVA22* family genes in tomatoes.

##  Supplemental Information

10.7717/peerj.14844/supp-1 Supplemental Information 1 Motif 1-20 sequences

10.7717/peerj.14844/supp-2 Table S1 RT-PCR primers of SpiHVA22s

10.7717/peerj.14844/supp-3 Table S2 CDS sequences of predicted candidate genes for four species *Lycopersicon*

10.7717/peerj.14844/supp-4 Table S3 *Lycopersicon* HVA22 protein sequences used for phylogenetic tree construction

10.7717/peerj.14844/supp-5 Table S4 Solanum HVA22 protein sequences used for phylogenetic tree construction

10.7717/peerj.14844/supp-6 Table S5 Predicted Interaction relationships between *HVA22* genes and Proteins

10.7717/peerj.14844/supp-7 Table S6 RPKM valuse of RNA-seq data in different tissues of *Solaunm pimpinellifolium*

10.7717/peerj.14844/supp-8 Table S7 qRT-PCR expression analysis data under abiotic stress

## Additional Information and Declarations

### Data Availability

The following information was supplied regarding data availability: The raw data is available in the Supplemental Files.

## References

[ref-1] Afrin T, Diwan D, Sahawneh K, Pajerowska-Mukhtar K (2020). Multilevel regulation of endoplasmic reticulum stress responses in plants: where old roads and new paths meet. Journal of Experimental Botany.

[ref-2] Artimo P, Jonnalagedda M, Arnold K, Baratin D, Csardi G, De Castro E, Duvaud S, Flegel V, Fortier A, Gasteiger E (2012). ExPASy: SIB bioinformatics resource portal. Nucleic Acids Research.

[ref-3] Brands A, Ho T-HD (2002). Function of a plant stress-induced gene, HVA22. Synthetic enhancement screen with its yeast homolog reveals its role in vesicular traffic. Plant Physiology.

[ref-4] Calero M, Whittaker GR, Collins RN (2001). Yop1p, the yeast homolog of the polyposis locus protein 1, interacts with Yip1p and negatively regulates cell growth. Journal of Biological Chemistry.

[ref-5] Chaudhary J, Alisha A, Bhatt V, Chandanshive S, Kumar N, Mir Z, Kumar A, Yadav SK, Shivaraj S, Sonah H (2019a). Mutation breeding in tomato: advances, applicability and challenges. Plants.

[ref-6] Chaudhary J, Khatri P, Singla P, Kumawat S, Kumari A, Vinaykumar R, Vikram A, Jindal SK, Kardile H, Kumar R, Sonah H, Deshmukh R (2019b). Advances in omics approaches for abiotic stress tolerance in tomato. Biology.

[ref-7] Chen C, Chen H, Zhang Y, Thomas HR, Frank MH, He Y, Xia R (2020). TBtools: an integrative toolkit developed for interactive analyses of big biological data. Molecular Plant.

[ref-8] Chen C-N, Chu C-C, Zentella R, Pan S-M, David Ho T-H (2002). AtHVA22 gene family in Arabidopsis: phylogenetic relationship, ABA and stress regulation, and tissue-specific expression. Plant Molecular Biology.

[ref-9] Chen H, Ma B, Zhou Y, He S-J, Tang S-Y, Lu X, Xie Q, Chen S-Y, Zhang J-S (2018). E3 ubiquitin ligase SOR1 regulates ethylene response in rice root by modulating stability of Aux/IAA protein. Proceedings of the National Academy of Sciences of the United States of America.

[ref-10] Cho SK, Ryu MY, Seo DH, Kang BG, Kim WT (2011). The Arabidopsis RING E3 ubiquitin ligase AtAIRP2 plays combinatory roles with AtAIRP1 in abscisic acid-mediated drought stress responses. Plant Physiology.

[ref-11] Collin A, Daszkowska-Golec A, Kurowska M, Szarejko I (2020). Barley ABI5 (Abscisic Acid INSENSITIVE 5) is involved in abscisic acid-dependent drought response. Frontiers in Plant Science.

[ref-12] De Antoni A, Schmitzová J, Trepte H-H, Gallwitz D, Albert ST (2002). Significance of GTP hydrolysis in Ypt1p-regulated endoplasmic reticulum to Golgi transport revealed by the analysis of two novel Ypt1-GAPs. Journal of Biological Chemistry.

[ref-13] De Bodt S, Maere S, Van de Peer Y (2005). Genome duplication and the origin of angiosperms. Trends in Ecology & Evolution.

[ref-14] Eggert E, Obata T, Gerstenberger A, Gier K, Brandt T, Fernie AR, Schulze W, Kühn C (2016). A sucrose transporter-interacting protein disulphide isomerase affects redox homeostasis and links sucrose partitioning with abiotic stress tolerance. Plant, Cell & Environment.

[ref-15] Fei Z, Joung J-G, Tang X, Zheng Y, Huang M, Lee JM, McQuinn R, Tieman DM, Alba R, Klee HJ (2010). Tomato functional genomics database: a comprehensive resource and analysis package for tomato functional genomics. Nucleic Acids Research.

[ref-16] Fernandez-Pozo N, Menda N, Edwards JD, Saha S, Tecle IY, Strickler SR, Bombarely A, Fisher-York T, Pujar A, Foerster H (2015). The Sol Genomics Network (SGN)—from genotype to phenotype to breeding. Nucleic Acids Research.

[ref-17] Finn RD, Clements J, Eddy SR (2011). HMMER web server: interactive sequence similarity searching. Nucleic Acids Research.

[ref-18] Gao C, Zhuang X, Shen J, Jiang L (2017). Plant ESCRT complexes: moving beyond endosomal sorting. Trends in Plant Science.

[ref-19] Gil K-E, Kim W-Y, Lee H-J, Faisal M, Saquib Q, Alatar AA, Park C-M (2017). ZEITLUPE contributes to a thermoresponsive protein quality control system in Arabidopsis. The Plant Cell.

[ref-20] Gomes Ferreira MD, Araújo Castro J, Santana Silva RJ, Micheli F (2019). HVA22 from citrus: a small gene family whose some members are involved in plant response to abiotic stress. Plant Physiology and Biochemistry.

[ref-21] Gong Z, Xiong L, Shi H, Yang S, Herrera-Estrella LR, Xu G, Chao DY, Li J, Wang PY, Qin F, Li J, Ding Y, Shi Y, Wang Y, Yang Y, Guo Y, Zhu JK (2020). Plant abiotic stress response and nutrient use efficiency. Science China Life Sciences.

[ref-22] Grundy WN, Bailey TL, Elkan CP, Baker ME (1997). Meta-MEME: motif-based hidden Markov models of protein families. Bioinformatics.

[ref-23] Grzesiak MT, Hordyńska N, Maksymowicz A, Grzesiak S, Szechyńska-Hebda M (2019). Variation among spring wheat (triticum aestivum l.) genotypes in response to the drought stress, II—Root system structure. Plants.

[ref-24] Guo WJ, David Ho TH (2008). An abscisic acid-induced protein, HVA22, inhibits gibberellin-mediated programmed cell death in cereal aleurone cells. Plant Physiology.

[ref-25] He F, Wang HL, Li HG, Su Y, Li S, Yang Y, Feng CH, Yin W, Xia X (2018). Pe CHYR 1, a ubiquitin E3 ligase from Populus euphratica, enhances drought tolerance via ABA-induced stomatal closure by ROS production in Populus. Plant Biotechnology Journal.

[ref-26] Hentze MW, Castello A, Schwarzl T, Preiss T (2018). A brave new world of RNA-binding proteins. Nature Reviews Molecular Cell Biology.

[ref-27] Hernandez-Garcia CM, Finer JJ (2014). Identification and validation of promoters and cis-acting regulatory elements. Plant Science.

[ref-28] Horton P, Park K-J, Obayashi T, Fujita N, Harada H, Adams-Collier C, Nakai K (2007). WoLF PSORT: protein localization predictor. Nucleic Acids Research.

[ref-29] Howell SH (2021). Evolution of the unfolded protein response in plants. Plant, Cell & Environment.

[ref-30] Hu J, Shibata Y, Voss C, Shemesh T, Li Z, Coughlin M, Kozlov MM, Rapoport TA, Prinz WA (2008). Membrane proteins of the endoplasmic reticulum induce high-curvature tubules. Science.

[ref-31] Huang Y-L, Zhang L-K, Zhang K, Chen S-M, Hu J-B, Cheng F (2022). The impact of tandem duplication on gene evolution in Solanaceae species. Journal of Integrative Agriculture.

[ref-32] Kaur A, Pati PK, Pati AM, Nagpal AK (2017). In-silico analysis of cis-acting regulatory elements of pathogenesis-related proteins of Arabidopsis thaliana and Oryza sativa. PLOS ONE.

[ref-33] Kim JY, Jang I-C, Seo HS (2016). COP1 controls abiotic stress responses by modulating AtSIZ1 function through its E3 ubiquitin ligase activity. Frontiers in Plant Science.

[ref-34] Knapp S (2012). The tomato genome sequence provides insights into fleshy fruit evolution. Nature.

[ref-35] Koops P, Pelser S, Ignatz M, Klose C, Marrocco-Selden K, Kretsch T (2011). EDL3 is an F-box protein involved in the regulation of abscisic acid signalling in Arabidopsis thaliana. Journal of Experimental Botany.

[ref-36] Letunic I, Bork P (2021). Interactive Tree Of Life (iTOL) v5: an online tool for phylogenetic tree display and annotation. Nucleic Acids Research.

[ref-37] Lin SS, Martin R, Mongrand S, Vandenabeele S, Chen KC, Jang IC, Chua NH (2008). RING1 E3 ligase localizes to plasma membrane lipid rafts to trigger FB1-induced programmed cell death in Arabidopsis. The Plant Journal.

[ref-38] Liu J-H, Zhou P, An Y, Wang Z, Du H, Huang B (2014). Characterization of gene expression associated with drought avoidance and tolerance traits in a perennial grass species. PLOS ONE.

[ref-39] Liu X, Afrin T, Pajerowska-Mukhtar KM (2019). Arabidopsis GCN2 kinase contributes to ABA homeostasis and stomatal immunity. Communications Biology.

[ref-40] Livak KJ, Schmittgen TD (2001). Analysis of relative gene expression data using real-time quantitative PCR and the 2^−ΔΔCT^ method. Methods.

[ref-41] Lu PL (2013). Physiological functional analysis of a stress-induced protein, HVA22, in *Escherichia coli*. Access Intternational Journals.

[ref-42] Luo H, Laluk K, Lai Z, Veronese P, Song F, Mengiste T (2010). The Arabidopsis Botrytis Susceptible1 Interactor defines a subclass of RING E3 ligases that regulate pathogen and stress responses. Plant Physiology.

[ref-43] Meng F, Zhao Q, Zhao X, Yang C, Liu R, Pang J, Zhao W, Wang Q, Liu M, Zhang Z (2022). A rice protein modulates endoplasmic reticulum homeostasis and coordinates with a transcription factor to initiate blast disease resistance. Cell Reports.

[ref-44] Mistry J, Chuguransky S, Williams L, Qureshi M, Salazar GA, Sonnhammer EL, Tosatto SC, Paladin L, Raj S, Richardson LJ (2021). Pfam: the protein families database in 2021. Nucleic Acids Research.

[ref-45] Paterson AH, Freeling M, Tang H, Wang X (2010). Insights from the comparison of plant genome sequences. Annual Review of Plant Biology.

[ref-46] Rombauts S, Déhais P, Van Montagu M, Rouzé P (1999). PlantCARE, a plant cis-acting regulatory element database. Nucleic Acids Research.

[ref-47] Rothan C, Diouf I, Causse M (2019). Trait discovery and editing in tomato. The Plant Journal.

[ref-48] Sato H, Tomita H, Nakazawa T, Wakana S, Tamai M (2005). Deleted in polyposis 1-like 1 gene (Dp1l1): a novel gene richly expressed in retinal ganglion cells. Investigative Ophthalmology & Visual Science.

[ref-49] Sharon K, Suvarna S (2017). Cloning of HVA22 homolog from *Aloe vera* and preliminary study of transgenic plant development. International Journal of Pure Applied Bioscience.

[ref-50] Shen Q, Chen CN, Brands A, Pan SM, Ho TH (2001). The stress- and abscisic acid-induced barley gene HVA22: developmental regulation and homologues in diverse organisms. Plant Molecular Biology.

[ref-51] Shen Q, Uknes S, Ho T (1993). Hormone response complex in a novel abscisic acid and cycloheximide-inducible barley gene. Journal of Biological Chemistry.

[ref-52] Shiu S-H, Bleecker AB (2003). Expansion of the receptor-like kinase/Pelle gene family and receptor-like proteins in Arabidopsis. Plant Physiology.

[ref-53] Shu K, Yang W (2017). E3 ubiquitin ligases: ubiquitous actors in plant development and abiotic stress responses. Plant and Cell Physiology.

[ref-54] Soltis PS, Soltis DE (2016). Ancient WGD events as drivers of key innovations in angiosperms. Current Opinion in Plant Biology.

[ref-55] Spitzer C, Reyes FC, Buono R, Sliwinski MK, Haas TJ, Otegui MS (2009). The ESCRT-related CHMP1A and B proteins mediate multivesicular body sorting of auxin carriers in Arabidopsis and are required for plant development. The Plant Cell.

[ref-56] Szklarczyk D, Gable AL, Lyon D, Junge A, Wyder S, Huerta-Cepas J, Simonovic M, Doncheva NT, Morris JH, Bork P (2019). STRING v11: protein–protein association networks with increased coverage, supporting functional discovery in genome-wide experimental datasets. Nucleic Acids Research.

[ref-57] Szymański J, Bocobza S, Panda S, Sonawane P, Cárdenas PD, Lashbrooke J, Kamble A, Shahaf N, Meir S, Bovy A (2020). Analysis of wild tomato introgression lines elucidates the genetic basis of transcriptome and metabolome variation underlying fruit traits and pathogen response. Nature Genetics.

[ref-58] Tamura K, Stecher G, Kumar S (2021). MEGA11: molecular evolutionary genetics analysis version 11. Molecular Biology and Evolution.

[ref-59] Thomas PD, Ebert D, Muruganujan A, Mushayahama T, Albou LP, Mi H (2022). PANTHER: making genome-scale phylogenetics accessible to all. Protein Science.

[ref-60] Voeltz GK, Prinz WA, Shibata Y, Rist JM, Rapoport TA (2006). A class of membrane proteins shaping the tubular endoplasmic reticulum. Cell.

[ref-61] Wai AH, Waseem M, Cho LH, Kim ST, Lee DJ, Kim CK, Chung MY (2022). Comprehensive genome-wide analysis and expression pattern profiling of the SlHVA22 gene family unravels their likely involvement in the abiotic stress adaptation of tomato. International Journal of Molecular Sciences.

[ref-62] Wang D, Zhang Y, Zhang Z, Zhu J, Yu J (2010). KaKs_Calculator 2.0: a toolkit incorporating gamma-series methods and sliding window strategies. Genomics, Proteomics & Bioinformatics.

[ref-63] Wang L, Zhu W, Fang L, Sun X, Su L, Liang Z, Wang N, Londo JP, Li S, Xin H (2014). Genome-wide identification of WRKY family genes and their response to cold stress in Vitis vinifera. BMC Plant Biology.

[ref-64] Wood TE, Takebayashi N, Barker MS, Mayrose I, Greenspoon PB, Rieseberg LH (2009). The frequency of polyploid speciation in vascular plants. Proceedings of the National Academy of Sciences of the United States of America.

[ref-65] Wu F, Tanksley SD (2010). Chromosomal evolution in the plant family Solanaceae. BMC Genomics.

[ref-66] Wu S, Han B, Jiao Y (2020). Genetic contribution of paleopolyploidy to adaptive evolution in angiosperms. Molecular Plant.

[ref-67] Xiang L, Etxeberria E, Ende WVanden (2013). Vacuolar protein sorting mechanisms in plants. The FEBS Journal.

[ref-68] Xu G, Guo C, Shan H, Kong H (2012). Divergence of duplicate genes in exon–intron structure. Proceedings of the National Academy of Sciences of the United States of America.

[ref-69] Yadav A, Fernández-Baca D, Cannon SB (2020). Family-specific gains and losses of protein domains in the legume and grass plant families. Evolutionary Bioinformatics.

[ref-70] Yates AD, Allen J, Amode RM, Azov AG, Barba M, Becerra A, Bhai J, Campbell LI, Carbajo Martinez M, Chakiachvili M (2022). Ensembl Genomes 2022: an expanding genome resource for non-vertebrates. Nucleic Acids Research.

[ref-71] Zhang Y, Wong C-H, Birnbaum RY, Li G, Favaro R, Ngan CY, Lim J, Tai E, Poh HM, Wong E (2013). Chromatin connectivity maps reveal dynamic promoter–enhancer long-range associations. Nature.

[ref-72] Zhao Y, Wang Q, Zhang Y, Zhang P, Jiang M (2021). BIP130 enhances salt tolerance through modulation of ABA synthesis and scavenging ROS in rice (Oryza sativa L.). Plant Growth Regulation.

