# Supplementary figures and images for: Genome-wide identification and analysis of the evolution and expression pattern of the HVA22 gene family in three wild species of tomatoes
Source: PeerJ. 2023 Feb 13;11:e14844. doi: 10.7717/peerj.14844 (PMC9933743; doi:10.7717/peerj.14844)

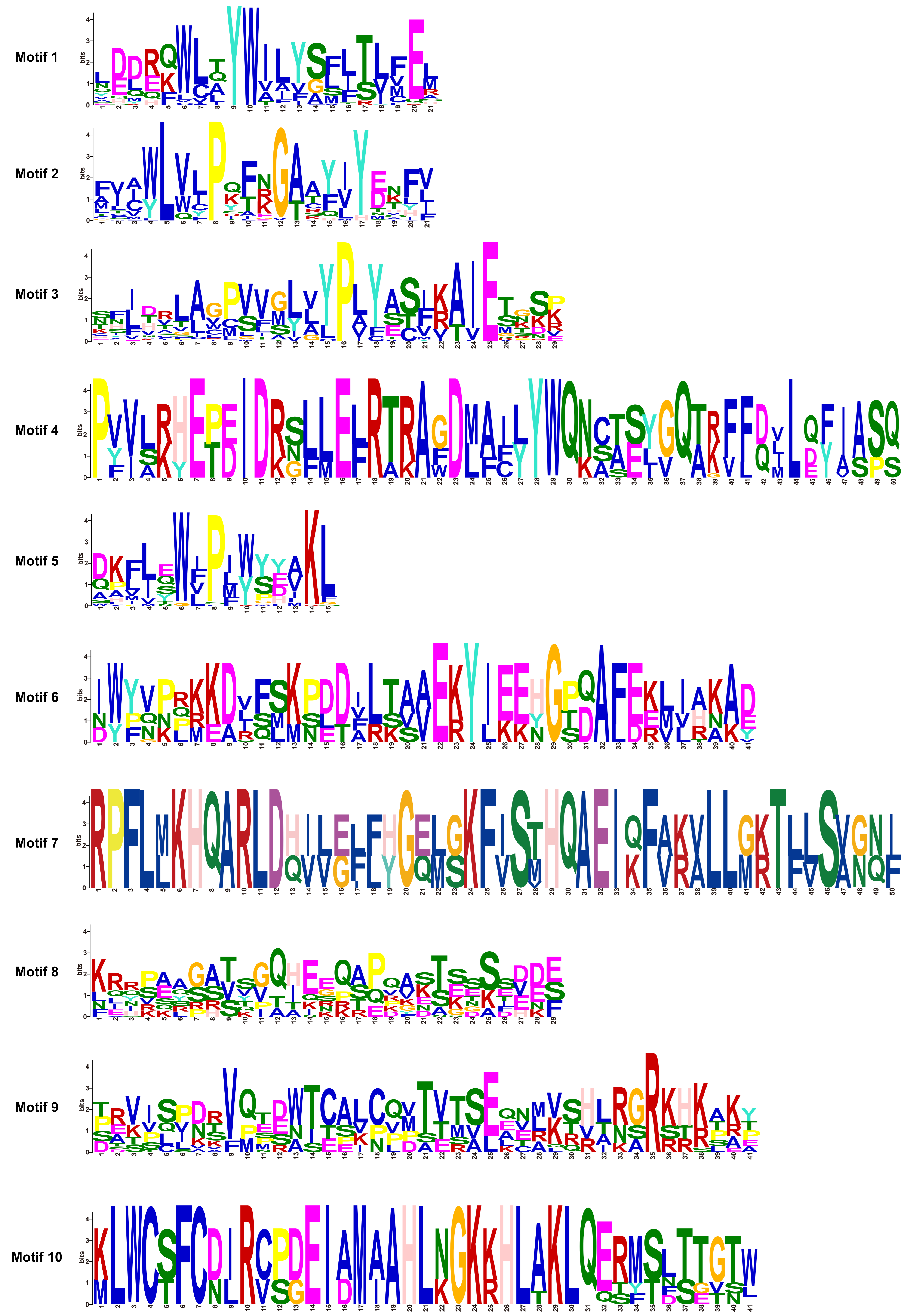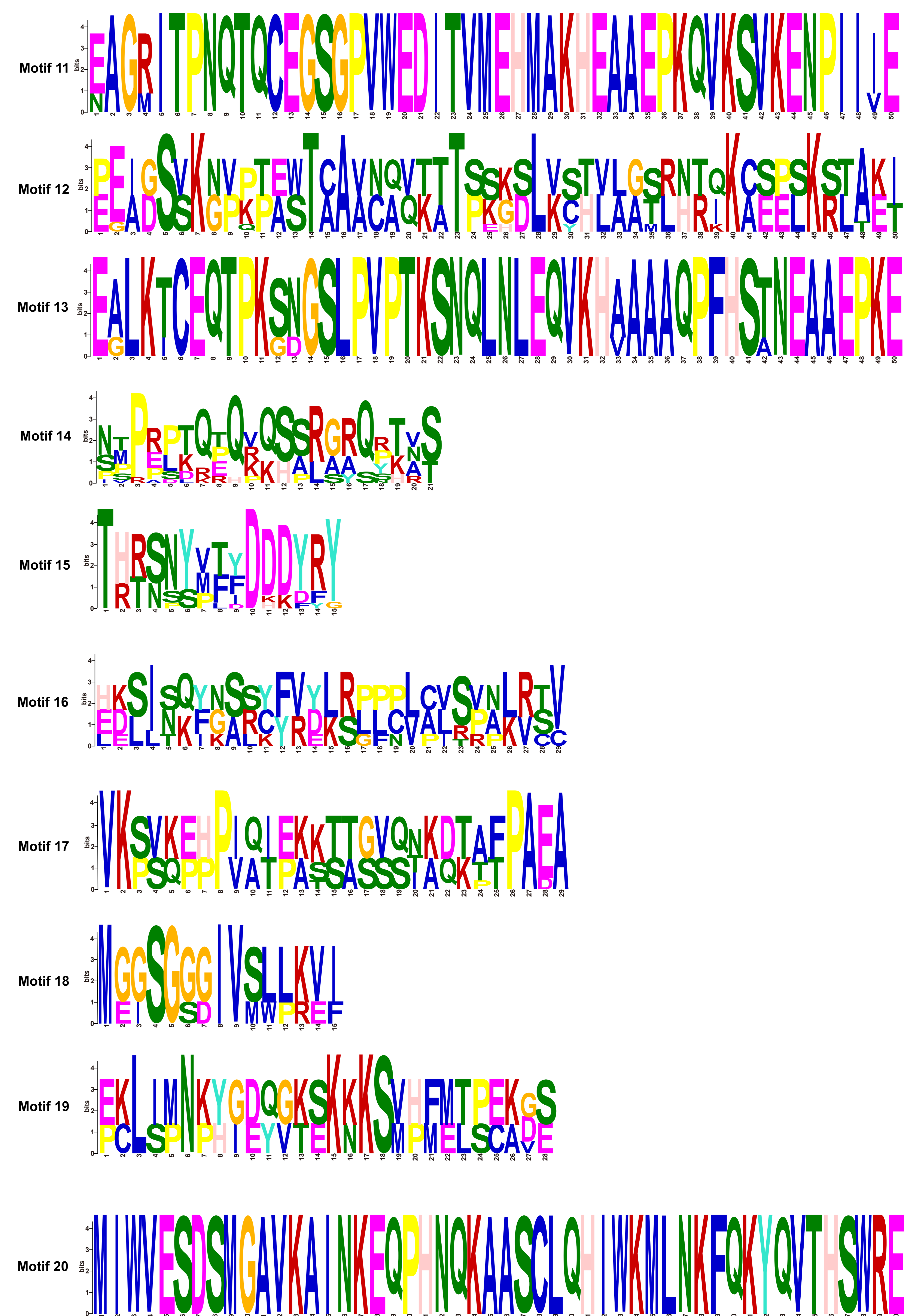

Supplement: Supplemental Information 1 [file peerj-11-14844-s001.pdf]
